# Stereoselective synthesis of fused tetrahydroquinazolines through one-pot double [3 + 2] dipolar cycloadditions followed by [5 + 1] annulation

**DOI:** 10.3762/bjoc.12.211

**Published:** 2016-10-18

**Authors:** Xiaofeng Zhang, Kenny Pham, Shuai Liu, Marc Legris, Alex Muthengi, Jerry P Jasinski, Wei Zhang

**Affiliations:** 1 Center for Green Chemistry and Department of Chemistry, University of Massachusetts Boston, 100 Morrissey Boulevard, Boston, MA 02125, USA; 2 Department of Chemistry, Keene State College, 220 Main Street, Keene, NH 03435, USA

**Keywords:** [5 + 1] annulation, [3 + 2] cycloaddition, one-pot reactions, stereoselective synthesis, tetrahydroquinazoline

## Abstract

The one-pot [3 + 2] cycloaddition of an azomethine ylide with a maleimide followed by another [3 + 2] cycloaddition of an azide with the second maleimide gives a 1,5-diamino intermediate which is used for a sequential aminomethylation reaction with formaldehyde through [5 + 1] annulation to afford a novel polycyclic scaffold bearing tetrahydroquinazoline, pyrrolidine, pyrrolidinedione, and *N*-substituted maleimide in stereoselective fashion.

## Introduction

**Scheme 1:**
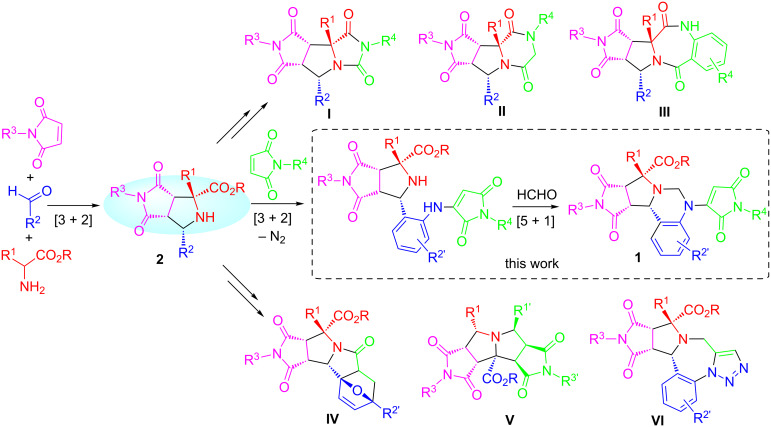
Polycyclic scaffolds derived from [3 + 2] adducts **2**.

**Figure 1:**
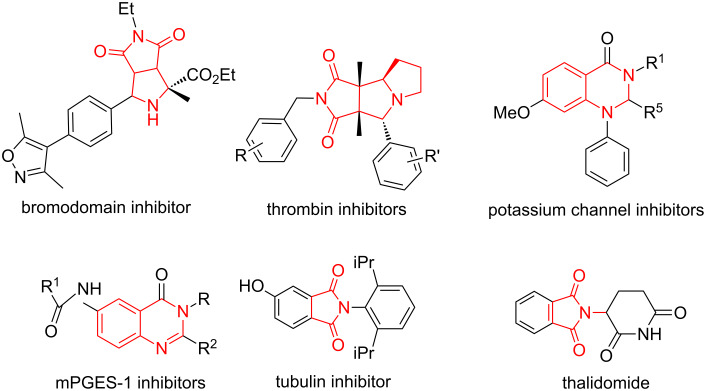
Heterocyclic fragments in bioactive compounds.

The synthesis of new molecules with potential biological activity through pot, atom and step-economic (PASE) reactions is an attractive green organic technique [1–5]. By the combination of multicomponent reactions (MCR) [6–11] with stepwise one-pot reactions [12–17], our lab has introduced a series of synthetic methods for heterocyclic compounds **I–VI** bearing heterocyclic rings such as hydantoin, pyrrolidine, pyrrolidinedione, piperazinedione, and dihydrobenzodiazepinedione (Scheme 1) [4,18–21]. All these scaffolds were prepared using one-pot intermolecular or intramolecular [3 + 2] azomethine ylide cycloadditions [22–27] as the initial step followed by cyclization or cycloaddition reactions to form polycyclic scaffolds with skeleton, substitution, and stereochemistry diversities. Introduced in this paper is a new sequence initiated with a three-component [3 + 2] cycloaddition for preparing polycyclic scaffold **1** bearing tetrahydroquinazoline, pyrrolidine, pyrrolidinedione, and *N*-substituted maleimide rings. Those heterocyclic fragments could be found in bioactive compounds such as bromodomain, thrombin, potassium channel, mPGES-1, and tubulin inhibitors, as well as the immunomodulatory drug thalidomide [28–32] (Figure 1).

## Results and Discussion

Our initial effort was focused on the development of reaction conditions for the one-pot double [3 + 2] cycloadditions. The first [3 + 2] cycloaddition of azomethine ylide was carried out using glycine methyl ester (**3a**), 2-azidobenzaldehyde (**4a**), and *N*-methylmaleimide (**5a**) as reactants [33]. After exploring the reactions with different temperatures, times, solvents, and bases, it was found that with a 1.2:1.1:1.0 ratio of **3a**:**4a**:**5a**, Et_3_N as a base, and MeCN as a solvent, the three-component reaction for **2a** was completed under microwave heating at 115 °C for 25 min. Without work-up, the reaction mixture was directly reacted with 1.0 equiv of *N*-benzylmaleimide (**6a**) under microwave heating at 125 °C for 25 min to give **7a** as a major diastereomer of a denitrogenation compound in 74% LC yield with a 39:1 dr (Table 1, entry 5). The diastereomer **7a** was isolated in 65% yield by preparative chromatography. The stereochemistry of the final product was established during the first [3 + 2] cycloaddition of the azomethine ylide which has been well reported in literature [22–27].

**Table 1:** One-pot double [3 + 2] cycloaddition for **7a**^a^.

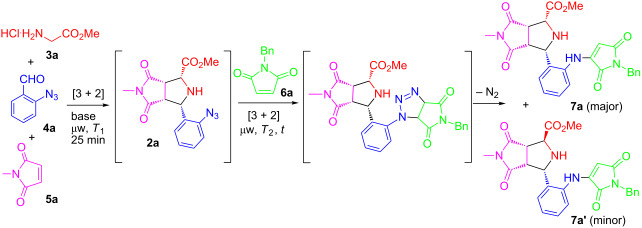							

entry	*T*_1_ (°C)	solvent	base (2 equiv)	*T*_2_ (°C)	*t* (min)	**7a** (%)^b^	dr

1	150	toluene	Et_3_N	150	25	65	40:1
2	125	dioxane	Et_3_N	125	25	33	15:1
3	115	EtOH	Et_3_N	115	25	45	21:1
4	115	CH_3_CN	Et_3_N	115	25	70	30:1
**5**	**115**	**CH_3_CN**	**Et_3_N**	**125**	**25**	**74 (65)^c^**	**39:1**
6	115	CH_3_CN	K_2_CO_3_	125	25	51	9:1
7	115	CH_3_CN	DBU	125	25	60	29:1
8	115	CH_3_CN	DIPEA	125	25	72	38:1
9	115	CH_3_CN	Et_3_N	125	10	63	35:1
10	115	CH_3_CN	Et_3_N	125	50	72	39:1
11	115	CH_3_CN	Et_3_N	150	25	68	41:1

^a^1.2:1.1:1.0:1.0 of **3a**:**4a**:**5a**:**6a**; ^b^detected by LC; ^c^isolated yield.

**Figure 2:**
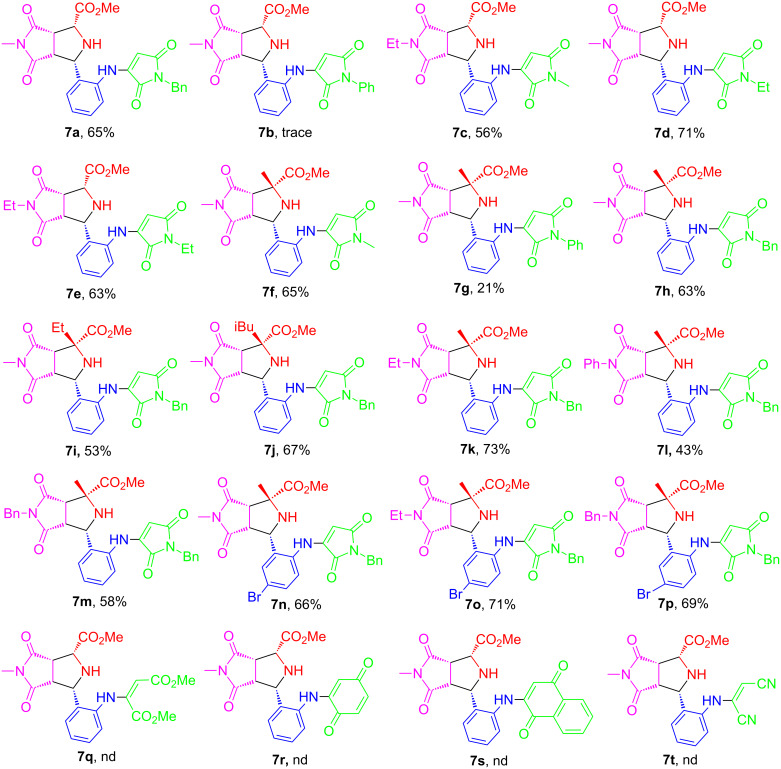
One-pot double [3 + 2] cycloadditions and denitrogenation for product **7** under the optimized reaction conditions, see Table 1, entry 5. nd = not detected.

We next explored the reaction scope of the one-pot double [3 + 2] reactions under the optimized conditions by using different sets of building blocks of **3**, **4**, **5**, and **6** to afford analogs **7a–p** in 21–73% isolated yields as single diastereomers (Figure 2). Compound **7b** was an exception, which was obtained in a trace amount. It was found that replacing maleimides **6** with other activated alkenes such as dimethyl maleate, benzoquinone, naphthalene-1,4-dione, and maleonitrile failed to afford products **7q**–**t,** probably due to unfavorable stereoelectronic effects associated with these substrates.

**Figure 3:**
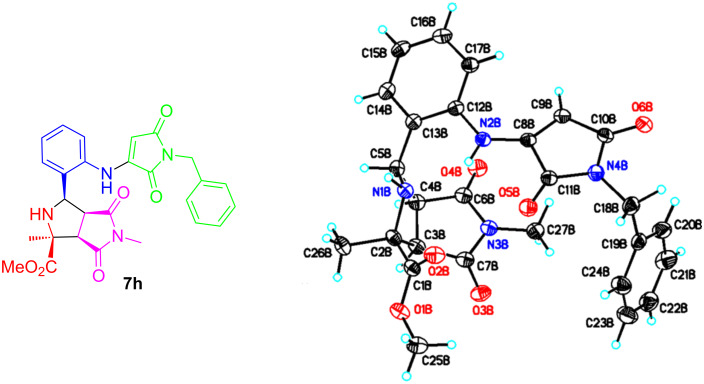
X-ray structure of **7h**.

**Scheme 2:**
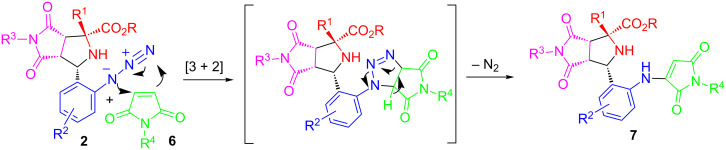
Proposed mechanism for the 2nd [3 + 2] cycloaddition and denitrogenation.

The stereochemistry of **7h** has been determined by X-ray single crystal structure analysis (Figure 3). As mentioned previously, the stereoselectivity of the first [3 + 2] cycloaddition for compounds **2** has been well reported [22–27]. The mechanism for the second [3 + 2] cycloaddition of azide compounds **2** with maleimides and sequential denitrogenation to products **7** is proposed in Scheme 2.

**Figure 4:**
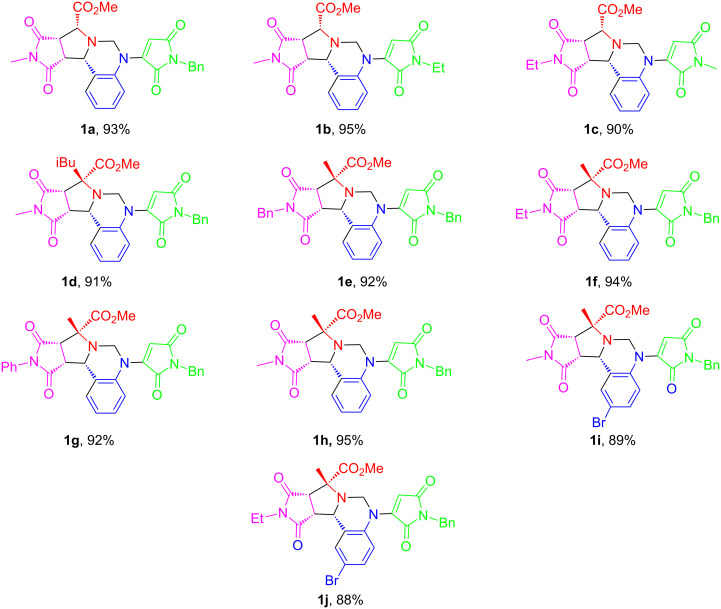
[5 + 1] Annulation for tetrahydroquinazolines **1**.

1,5-Diamino compounds **7** generated by one-pot reactions are good substrates for [5 + 1] annulation with aldehydes to form tetrahydroquinazolines **1** [34–35]. After exploring the reaction conditions, it was found that the reaction of **7a** with formaldehyde in 1,4-dioxane at 110 °C afforded product **1a** in 93% isolated yield (Table 2, entry 3). Other reactants such as HC(OEt)_3_, HCO_2_H, and paraformaldehyde (PFA) were also employed for the [5 + 1] annulation reactions. But only formaldehyde afforded tetrahydroquinazoline **1a** in good yield under catalyst-free conditions. A number of [5 + 1] annulation reactions using selected compounds **7** were carried out to afford 10 analogs of tetrahydroquinazolines **1** in 88–95% isolated yields as single diastereomers (Figure 4). In addition to formaldehyde, other aldehydes could also be used for the [5 + 1] annulation according to literature [34–35].

**Table 2:** Optimization of [5 + 1] annulation for product **1a**^a^.

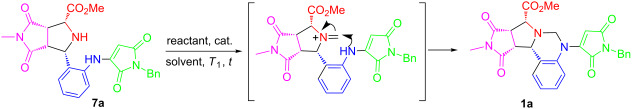						

entry	reactant (equiv)	catalyst (equiv)	solvent	*T*_1_ (°C)	*t* (h)	**1a** (%)^a^

1	HC(OEt)_3_ (1.5)	NH_4_Cl (2.0)	H_2_O	100	3	51
2	HCO_2_H (3.0)	–	H_2_O	100	5	nd^b^
**3**	**HCHO (3.0)**	**–**	**1,4-dioxane**	**110**	**3**	**93**
4	PFA (2.0)	TFA (3.0)	1,4-dioxane	110	4	73

^a^Isolated yield; ^b^nd = not detected.

## Conclusion

A one-pot reaction sequence involving [3 + 2] cycloaddition of azomethine ylides, [3 + 2] cycloaddition of azides with alkenes, and denitrogenation followed by a [5 + 1] anulation has been developed for the synthesis of fused-tetrahydroquinazolines as single diastereomers. The formation of triazoles from the second [3 + 2] cycloaddition readily affords denitrogenated 1,5-diamino compounds which are good substrates for aminomethylation with formaldehyde through a [5 + 1] annulation. The final products have a unique polycyclic skeleton containing tetrahydroquinazoline, pyrrolidine, pyrrolidinedione, and *N*-substituted maleimide ring systems.

## Experimental

### General Information

Chemicals and solvents were purchased from commercial suppliers and used as received. ^1^H NMR (300 or 400 MHz) and ^13^C NMR spectra (75 or 101 MHz) were recorded on Agilent NMR spectrometers. Chemical shifts were reported in parts per million (ppm), and the residual solvent peak was used as an internal reference: proton (chloroform δ 7.26; dioxane δ 3.71; H_2_O δ 1.56), carbon (chloroform δ 77.0). Multiplicity was indicated as follows: s (singlet), d (doublet), t (triplet), q (quartet), m (multiplet), dd (doublet of doublet), br s (broad singlet). Coupling constants were reported in hertz (Hz). LC–MS was performed on an Agilent 2100 LC with a 6130 quadrupole MS spectrometer. A C18 column (5.0 μm, 6.0 × 50 mm) was employed for the separation. The mobile phases were MeOH and H_2_O both of which contained 0.05% CF_3_CO_2_H. A linear gradient from 25:75 (v/v) MeOH/water to 100% MeOH over 7.0 min at a flow rate of 0.7 mL/min was employed as a mobile phase. UV detections were conducted at 210 nm, 254 nm and 365 nm. Low resolution mass spectra were recorded with APCI (atmospheric pressure chemical ionization). The final products were purified on Angela HP-100 pre-LC system with a Venusil PrepG C18 column (10 μm, 120 Å, 21.2 mm × 250 mm).

### General procedure for the one-pot synthesis of compounds **7**

The following procedure is analogous to one of our previous procedures [4]. To a solution of an amino ester **3** (1.2 mmol), 2-azidobenzaldehyde (**4**, 1.1 mmol), and maleimide **5** (1.0 mmol) in 2.0 mL of CH_3_CN was added Et_3_N (2.0 mmol). After being stirred at 25 °C for 5 min, the reaction mixture was heated by microwave irradiation at 115 °C for 25 min. Upon the completion of the reaction as monitored by LC–MS, maleimide **6** (1.0 mmol) was added to the reaction mixture and then heated by microwave irradiation at 125 °C for 25 min. The concentrated reaction mixture was isolated on a semi-preparative HPLC with a C18 column to afford purified product **7** as a single diastereomer.

### General procedure for [5 + 1] annulation for the synthesis of products **1**

To a solution of compound **7** (0.5 mmol), in 1.0 mL of 1,4-dioxane was added formaldehyde solution (16% in H_2_O, 1.5 mmol). The reaction mixture was heated at 110 °C for 3 h. Upon the completion of the reaction as monitored by LC–MS, the reaction mixture was concentrated and then isolated on a semi-preparative HPLC with a C18 column to afford purified product **1**.

## Supporting Information

File 1 Compound characterization data, X-ray report, and copies of NMR spectra.
