# Supplementary material for: Stereoselective synthesis of fused tetrahydroquinazolines through one-pot double [3 + 2] dipolar cycloadditions followed by [5 + 1] annulation
Source: Beilstein J Org Chem. 2016 Oct 18;12:2204–10. doi: 10.3762/bjoc.12.211 (PMC5082442; doi:10.3762/bjoc.12.211)
Supplement: File 1 — Compound characterization data, X-ray report, and copies of NMR spectra. [file Beilstein_J_Org_Chem-12-2204-s001.pdf]

Supporting Information
for

Stereoselective synthesis of fused tetrahydroquinazolines through one-pot double [3 + 2] dipolar cycloadditions followed by [5 + 1] annulation

Xiaofeng Zhang[1], Kenny Pham[1], Shuai Liu[1], Marc Legris[1], Alex Muthengi[1], Jerry P. Jasinski[2] and Wei Zhang[*1]

Addresses: [1]Center for Green Chemistry and Department of Chemistry, University of Massachusetts Boston, 100 Morrissey Boulevard, Boston, MA 02125, USA and [2]Department of Chemistry, Keene State College, 220 Main Street, Keene, NH 03435, USA

Email: Wei Zhang - wei2.zhang@umb.edu

*Corresponding author

Compound characterization data, X-ray report, and copies of NMR spectra

1. Compound characterization data ………………………………………...S2
2. X-ray report of **7h**…..…………………………………………………….....S6
3. NMR spectra of intermediates (selective) **7** and products **1**…………...S7

1. Compound characterization data

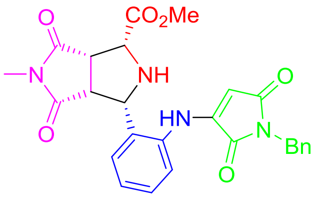

7a: 65% yield. ^{1}H NMR (300 MHz, $CDCl_3$) δ 9.88 (s, 1H), 7.41 – 6.93 (m, 9H), 5.37 (s, 1H), 4.66 – 4.49 (m, 3H), 4.09 (dd, J = 7.5, 4.1 Hz, 1H), 3.91 (s, 3H), 3.55 (t, J = 7.7 Hz, 1H), 3.35 (t, J = 8.4 Hz, 1H), 3.19 (s, 1H), 2.42 (s, 3H). ^{13}C NMR (75 MHz, $CDCl_3$) δ 174.5, 173.7, 172.3, 169.3, 167.2, 142.4, 139.0, 136.6, 129.9, 129.4, 128.5, 127.6, 125.1, 124.2, 119.0, 89.3, 64.5, 62.0, 52.4, 47.8, 46.6, 41.0, 24.6. APCIMS m/z: 489.2 (M^+ + 1)

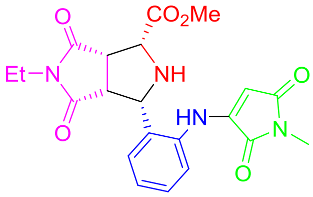

7c: 56% yield. ^{1}H NMR (300 MHz, $CDCl_3$) δ 10.03 (s, 1H), 7.37 – 6.97 (m, 4H), 5.32 (s, 1H), 4.56 (dd, J = 8.8, 4.0 Hz, 1H), 4.12 (dd, J = 7.4, 4.1 Hz, 1H), 3.91 (s, 3H), 3.60 – 3.26 (m, 3H), 3.26 – 3.01 (m, 2H), 2.89 (d, J = 4.0 Hz, 3H), 0.91 (t, J = 7.2 Hz, 3H). ^{13}C NMR (75 MHz, $CDCl_3$) δ 174.8, 173.8, 173.0, 169.9, 167.9, 142.4, 139.2, 129.7, 129.5, 124.2, 118.8, 89.5, 64.69, 62.2, 52.5, 51.1, 48.1, 46.8, 33.9, 23.8, 12.8. APCIMS m/z: 427.2 (M^+ + 1).

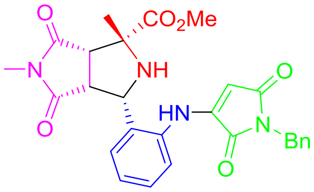

7h: 63% yield. ^{1}H NMR (300 MHz, $CDCl_3$) δ 9.43 (s, 1H), 7.43 – 7.01 (m, 9H), 5.39 (s, 1H), 4.80 – 4.53 (m, 3H), 3.87 (d, J = 16.6 Hz, 3H), 3.49 – 3.36 (m, 1H), 3.21 (d, J = 8.0 Hz, 1H), 2.47 (d, J = 6.2 Hz, 4H), 1.69 (d, J = 10.2 Hz, 3H). ^{13}C NMR (75 MHz, $CDCl_3$) δ 174.4, 173.8, 172.2, 171.7, 167.2, 143.3, 138.9, 136.7, 129.8, 129.3, 128.5, 127.7, 126.4, 124.6, 120.1, 89.5, 67.0, 61.6, 53.7, 52.7, 47.9, 41.1, 24.6, 23.4. APCIMS m/z: 503.2 (M^+ + 1).

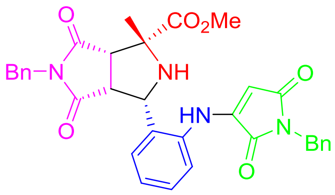

7m: 58% yield. ^{1}H NMR (300 MHz, $CDCl_3$) δ 9.21 (s, 1H), 7.43 – 7.03 (m, 15H), 5.43 (s, 1H), 4.76 – 4.62 (m, 2H), 4.08 (q, J = 13.7 Hz, 2H), 3.91 (s, 3H), 3.47 (d, J = 9.6 Hz, 1H), 3.20 (d, J = 7.9 Hz, 1H), 2.39 (d, J = 4.9 Hz, 1H), 1.64 (s, 3H). ^{13}C NMR

(75 MHz, $CDCl_3$) δ 174.0, 173.5, 172.3, 171.7, 167.2, 143.7, 138.5, 136.6, 135.0, 129.6, 128.8, 128.7, 128.0, 127.8, 127.1, 124.9, 120.5, 89.3, 66.9, 60.8, 53.9, 52.7, 47.8, 42.2, 41.2, 23.5. APCIMS m/z: 579.3 (M^+ + 1).

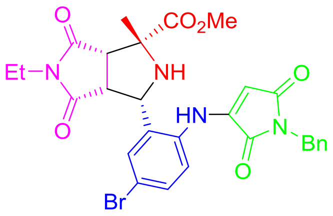

7o: 71% yield. ^{1}H NMR (400 MHz, $CDCl_3$) δ 8.96 (s, 1H), 7.52 – 6.99 (m, 8H), 5.34 (s, 1H), 4.73 – 4.51 (m, 3H), 3.87 (s, 3H), 3.51 – 3.33 (m, 1H), 3.19 (dd, J = 7.9, 1.0 Hz, 1H), 3.11 – 2.77 (m, 3H), 1.63 (s, 3H), 0.77 (t, J = 7.2 Hz, 3H). ^{13}C NMR (101 MHz, $CDCl_3$) δ 174.1, 173.5, 171.9, 171.6, 167.1, 143.4, 137.7, 136.5, 132.3, 132.1, 129.6, 128.6, 127.7, 121.9, 117.8, 90.1, 66.9, 60.2, 53.6, 52.6, 47.7, 41.2, 33.8, 23.6, 12.6. APCIMS m/z: 595.2 (M^+ + 1).

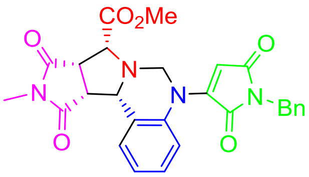

1a: 93% yield. ^{1}H NMR (400 MHz, $CDCl_3$) δ 7.42–7.18 (m, 8H), 7.05–6.92 (m, 2H), 4.64 (s, 2H), 4.31 (d, J = 8.5 Hz, 1H), 3.90 (s, 3H), 3.81–3.66 (m, 1H), 3.52 (dd, J = 27.7, 11.8 Hz, 2H), 3.33 (dd, J = 27.4, 7.9 Hz, 2H), 3.09 (s, 3H). ^{13}C NMR (101 MHz, $CDCl_3$) δ 176.3, 175., 172.3, 168.8, 166.3, 145.8, 138.1, 136.2, 132.0, 130.0, 128.6, 128.4, 127.8, 126.8, 125.2, 122.7, 94.7, 67.0, 64.6, 61.9, 52.4, 46.8, 45.2, 41.7, 37.4, 25.4. APCIMS m/z: 501.2 (M^+ + 1).

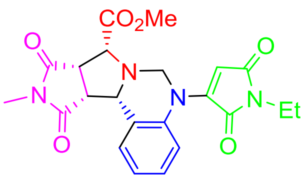

1b: 95% yield. ^{1}H NMR (400 MHz, $CDCl_3$) δ 7.46–7.17 (m, 3H), 7.12–6.93 (m, 2H), 4.32 (d, J = 8.5 Hz, 1H), 3.89 (s, 3H), 3.81–3.63 (m, 1H), 3.66–3.42 (m, 4H), 3.33 (dt, J = 14.1, 7.5 Hz, 2H), 3.09 (s, 3H), 1.30–1.05 (m, 3H). ^{13}C NMR (101 MHz, $CDCl_3$) δ 176.4, 175.1, 172.5, 168.8, 166.5, 145.7, 138.2, 132.0, 130.0, 126.7, 125.2, 122.6, 94.5, 64.6, 61.9, 52.4, 46.8, 45.2, 37.4, 33.1, 25.4, 13.9. APCIMS m/z: 439.3 (M^+ + 1).

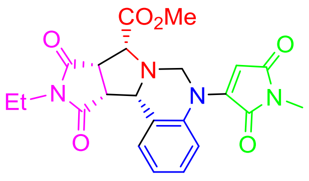

1c: 90% yield. ^{1}H NMR (400 MHz, $CDCl_3$)δ 7.39 – 7.19 (m, 3H), 7.12 – 6.88 (m, 2H), 4.32 (d, J = 8.5 Hz, 1H), 3.91 (d, J = 16.2 Hz, 3H), 3.69 (dt, J = 13.0, 11.4 Hz, 3H), 3.60 – 3.40 (m, 2H), 3.33 (dd,

J = 16.3, 7.9 Hz, 2H), 3.02 (d, J = 14.9 Hz, 3H), 1.24 (t, J = 7.1 Hz, 3H). ^{13}C NMR (101 MHz, $CDCl_3$) δ 176.1, 174.9, 172.7, 168.8, 166.7, 145.8, 138.2, 132.0, 130.0, 126.7, 125.4, 122.6, 94.7, 64.6, 61.9, 52.3, 46.7, 45.2, 37.4, 34.4, 24.0, 12.9. APCIMS m/z: 439.2 (M^+ + 1).

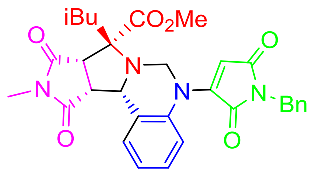

1d: 91% yield. ^{1}H NMR (400 MHz, $CDCl_3$) δ 7.43–7.05 (m, 7H), 6.92 (dd, J = 46.8, 7.5 Hz, 3H), 5.28 (d, J = 10.3 Hz, 1H), 4.65 (s, 2H), 3.89 – 3.60 (m, 3H), 3.60–3.44 (m, 3H), 3.30 (d, J = 14.4 Hz, 1H), 3.11 (s, 3H), 1.52–1.35 (m, 2H), 1.30–1.19 (m, 1H), 0.93 (dd, J = 37.2, 6.6 Hz, 6H). ^{13}C NMR (101 MHz $CDCl_3$) δ 175.9, 175.2, 174.0, 173.2, 171.7, 166.5, 138.5, 136.4, 131.7, 129.5, 128.5, 127.7, 125.9, 122.9, 99.2, 72.2, 67.0, 59.4, 52.3, 51.5, 47.1, 43.9, 41.6, 35.5, 25.2, 24.6, 23.4. APCIMS m/z: 557.3 (M^+ + 1).

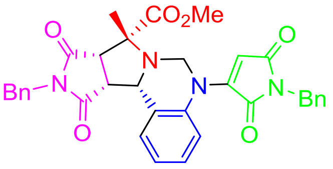

1e: 92% yield. ^{1}H NMR (400 MHz, $CDCl_3$) δ 7.59–7.05 (m, 11H), 7.05–6.85 (m, 2H), 6.85 – 6.63 (m, 1H), 6.45 (dd, J = 7.9, 1.0 Hz, 1H), 4.83 (dd, J = 11.8, 8.7 Hz, 2H), 4.71–4.51 (m, 3H), 3.85 (s, 3H), 3.67 (d, J = 16.5 Hz, 1H), 3.51–3.26 (m, 2H), 3.22 (d, J = 8.2 Hz, 1H), 1.34 (s, 3H). ^{13}C NMR (101 MHz,$CDCl_3$) δ 175.7, 174.7, 172.2, 171., 166.6, 144.4, 137.9, 136.3, 134.9, 132.0, 129.4, 128.6, 128.1, 127.7, 126.9, 126.3, 122.2, 99.0, 69.2, 58.5, 55.1, 52.3, 45.8, 42.9, 41.7, 36.0, 17.1. APCIMS m/z: 591.2 (M^+ + 1).

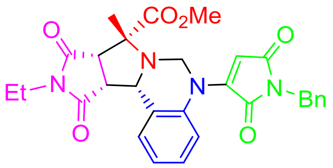

1f: 94% yield. ^{1}H NMR (400 MHz, $CDCl_3$) δ 7.45–7.10 (m, 7H), 7.07–6.85 (m, 3H), 4.88 (d, J = 9.8 Hz, 1H), 4.71–4.51 (m, 2H), 3.84 (s, 3H), 3.78–3.53 (m, 3H), 3.51 – 3.42 (m, 2H), 3.20 (d, J = 8.2 Hz, 1H), 1.36 (s, 3H), 1.26 (t, J = 7.2 Hz, 3H).^{13}C NMR (101 MHz, $CDCl_3$) δ 176.1, 174.9, 172.2, 171.5, 166.7, 144.4, 138.1, 136.3, 131.7, 129.6, 128.6, 128.2, 127.7, 127.0, 126.3, 122.4, 99.1, 69.1, 58.4, 55.0, 52.3, 45.8, 41.7, 36.1, 34.3, 17.1, 12.8. APCIMS m/z: 529.2 (M^+ + 1).

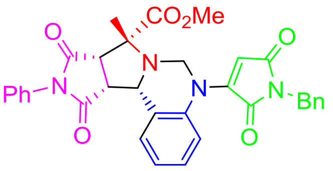

1g: 92% yield. ^{1}H NMR (400 MHz, $CDCl_3$) δ 7.56 – 6.90 (m, 15H), 4.99 (d, J = 9.9 Hz, 1H), 4.65 (d, J = 2.6 Hz, 2H), 3.81 (d, J = 17.9 Hz, 3H), 3.73 – 3.31 (m, 4H), 1.41 (s, 3H). ^{13}C NMR (101

MHz, $CDCl_3$) δ 175.0, 174.1, 172.2, 171.5, 144.4, 138.1, 136.3, 131.7, 129.6, 129.3, 128.7, 128.2, 127.8, 126.9, 126.6, 126.1, 122.5, 99.1, 69.4, 58.9, 55.0, 52.4, 45.9, 41.7, 36.1, 17.2. APCIMS m/z: 577.3 (M^+ + 1).

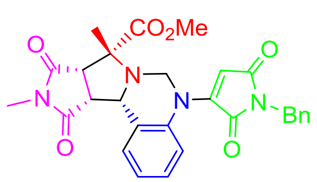

1h: 95% yield. ^{1}H NMR (400 MHz, $CDCl_3$) δ 7.25 (ddd, J = 25.7, 13.7, 5.8 Hz, 7H), 7.07– 6.94 (m, 2H), 6.87 (d, J = 7.8 Hz, 1H), 4.88 (d, J = 9.9 Hz, 1H), 4.65 (t, J = 8.9 Hz, 2H), 3.84 (s, 3H), 3.75 (d, J = 16.5 Hz, 1H), 3.45 (dd, J = 17.7, 9.9 Hz, 2H), 3.22 (d, J = 8.0 Hz, 1H), 3.08 (s, 3H), 1.35 (s, 3H). ^{13}C NMR (101 MHz, $CDCl_3$) δ 176.3, 175.1, 172.2, 171.5, 166.7, 144.5, 138.1, 136.3, 131.4, 129.6, 128.6, 128.1, 127.7, 126.8, 126.5, 122.4, 99.1, 69.1, 58.3, 55.1, 52.3, 45.8, 41.7, 36.0, 25.2, 17.0. APCIMS m/z: 515.2 (M^+ + 1).

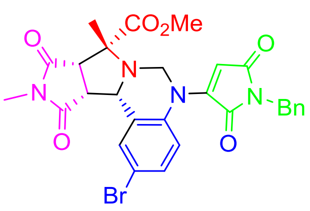

1i: 89% yield. ^{1}H NMR (400 MHz, $CDCl_3$) δ 7.59–7.05 (m, 7H), 6.89 (dd, J = 11.9, 5.2 Hz, 2H), 4.81 (d, J = 9.9 Hz, 1H), 4.69 – 4.49 (m, 2H), 3.91–3.71 (m, 3H), 3.48 (dd, J = 17.2, 11.3 Hz, 2H), 3.24 (d, J = 8.0 Hz, 1H), 3.11 (s, 3H), 1.34 (s, 3H). ^{13}C NMR (101 MHz $CDCl_3$) δ 176.0, 174.8, 171.9, 171.4, 166.5, 144.0, 137.2, 136.2, 134.8, 132.5, 129.0, 128.6, 128.2, 127.8, 123.7, 119.6, 99.3, 69.0, 58.1, 55.0, 52.4, 45.8, 41.8, 36.0, 25.1, 17.0. APCIMS m/z: 593.1 (M^+ + 1).

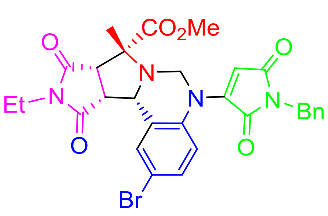

1j: 88% yield. ^{1}H NMR (400 MHz, $CDCl_3$) δ 7.39 (dd, J = 8.5, 2.2 Hz, 1H), 7.35–7.19 (m, 6H), 6.97 (s, 1H), 6.89 (d, J = 8.5 Hz, 1H), 4.81 (d, J = 9.9 Hz, 1H), 4.70–4.58 (m, 2H), 3.85 (s, 3H), 3.76 (d, J = 16.5 Hz, 1H), 3.50–3.41 (m, 4H), 3.20 (d, J = 8.2 Hz, 1H), 1.40–1.24 (m, 6H). ^{13}C NMR (101 MHz $CDCl_3$) δ 175.7, 174.7, 172.0, 171.4, 166.5, 144.0, 137.2, 136.2, 134.7, 132.6, 129.1, 128.6, 128.2, 127.8, 123.8, 119.6, 99.4, 69.1, 58.1, 54.9, 52.4, 45.8, 41.7, 36.0, 34.4, 17.2, 13.0. APCIMS m/z: 607.2 (M^+ + 1).

2. X-ray report of 7h

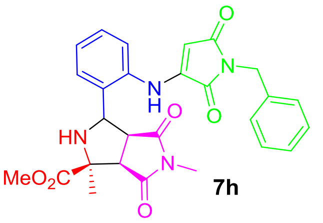

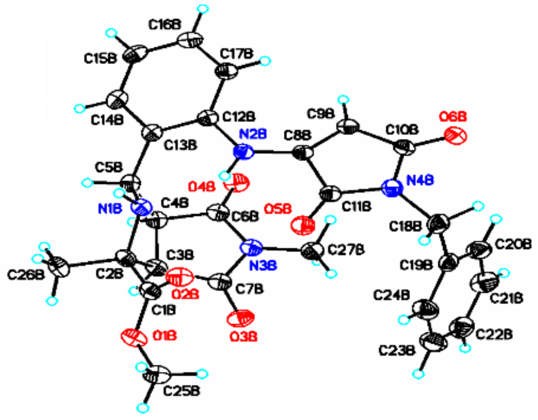

Bond precision	c-c=0.0026 Å	Wavelength=1.54184
Cell	a=11.0454 (5) b=14.1639(7) c=16.6788(8)	
	α=94.288(4) β=102.590(4) γ=99.650(4)	
Temperature	173K	
	Calculated	Reported
Volumn	2494.0(2)	2494.0(2)
Space group	P -1	P -1
Hall group	-P 1	-P 1
Moiety formula	C_{27} H_{26} N_4 O_6	C_{27} H_{26} N_4 O_6
Sum formula	C_{27} H_{26} N_4 O_6	C_{27} H_{26} N_4 O_6
Mr	502.52	502.52
Dx,g cm^{-3}	1.338	1.338
Z	4	4
Mu (mm^{-1})	0.796	0.796
F000	1056.0	1056.0
F000’	1059.4	
h,k,lmax	13, 17, 20	13, 17, 20
Nref	9714	9482
Tmin,Tmax	0.810, 0.880	0.935, 1.000
Tmin’	0.739	
Correction method= Multi-scan		
AbsCorr = MULTI-SCAN		
Data completeness= 0.976	Theta(max)= 71.359	
R(reflections)= 0.0448(7584)	wR2(reflections)= 0.1245(9482)	
S = 1.038	Npar= 681	

Crystallographic data (excluding structural factors) for compound **7h** has been deposited at the Cambridge Crystallographic Data Centre under the deposit ion number CCDC1497990.

3. NMR spectra of intermediates 7 (selective) and products 1

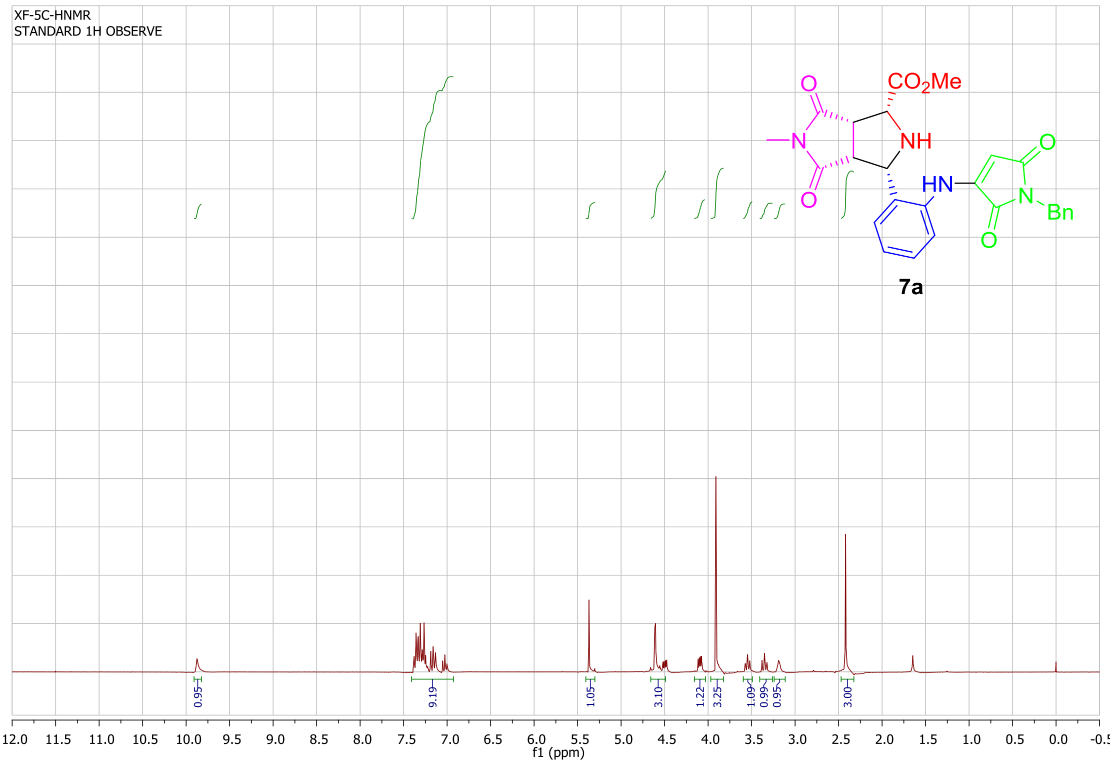

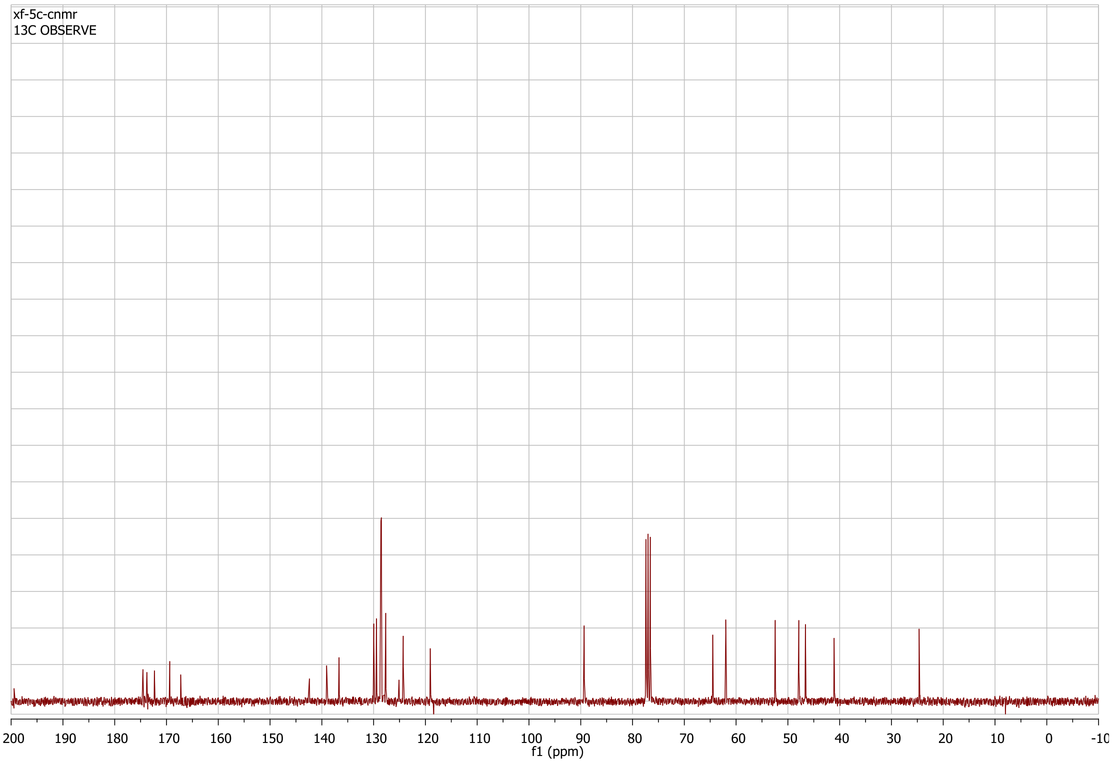

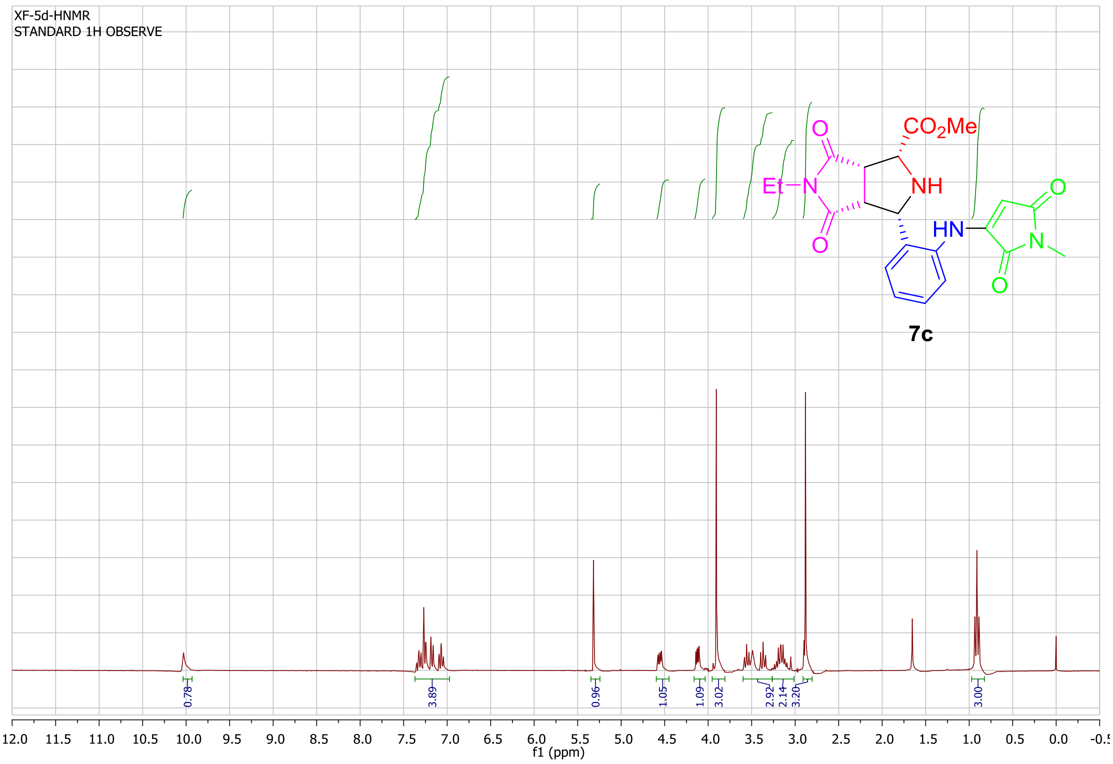
XF-5d-HNMR
STANDARD 1H OBSERVE
CO2Me
O
Et
N
NH
HN
O
N
O
O
7c
0.78
3.89
0.96
1.05
1.09
3.02
2.92
2.14
3.20
3.00
f1 (ppm)

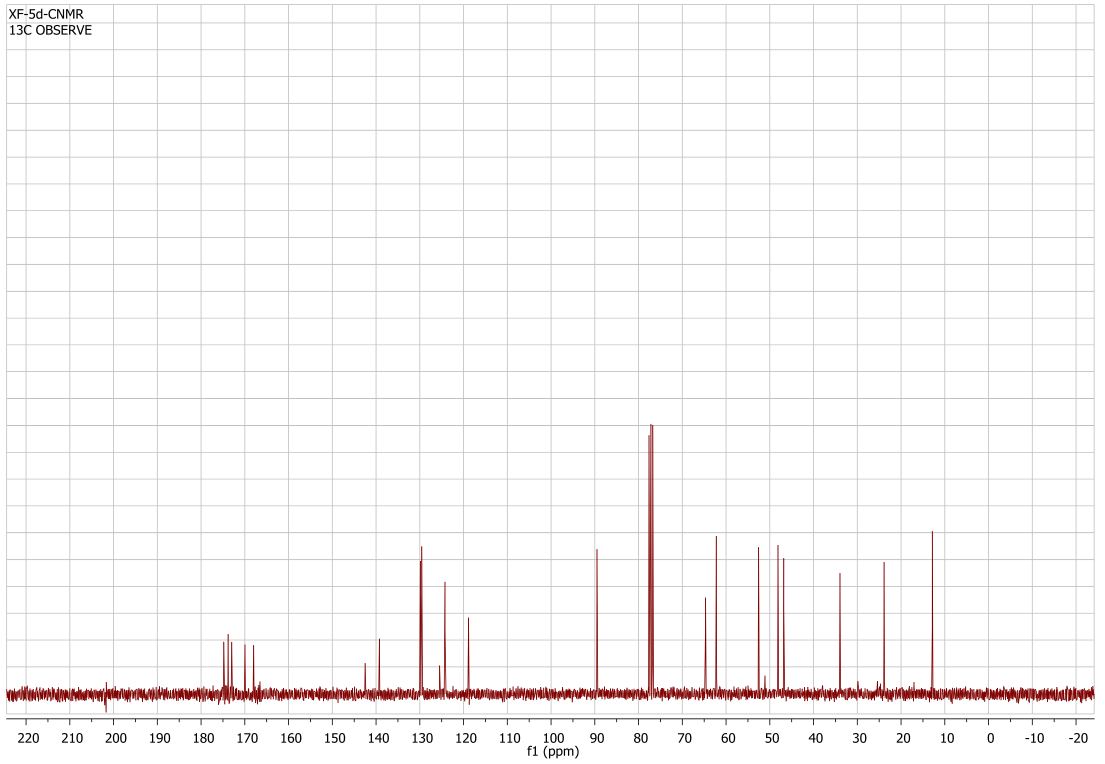
XF-5d-CNMR
13C OBSERVE
f1 (ppm)

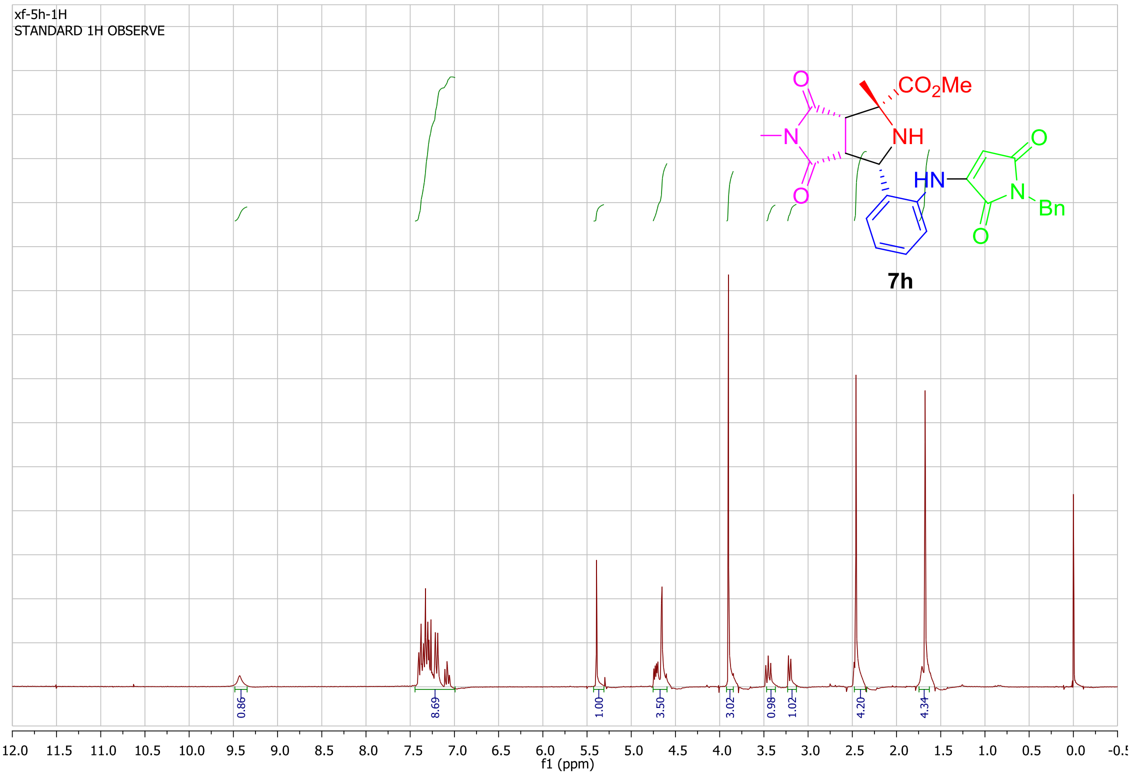
xf-5h-1H
STANDARD 1H OBSERVE
CO2Me
NH
HN
N
Bn
O
7h
0.86
8.69
1.00
3.50
3.02
0.98
1.02
4.20
4.34
f1 (ppm)

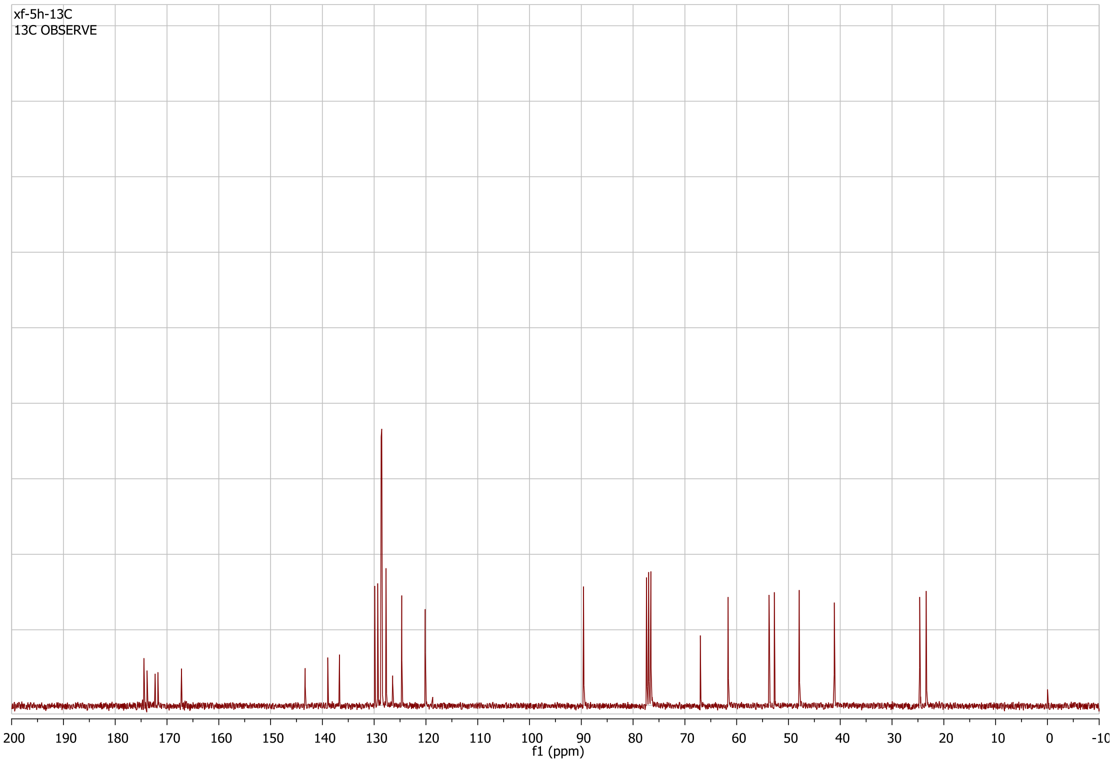
xf-5h-13C
13C OBSERVE
f1 (ppm)

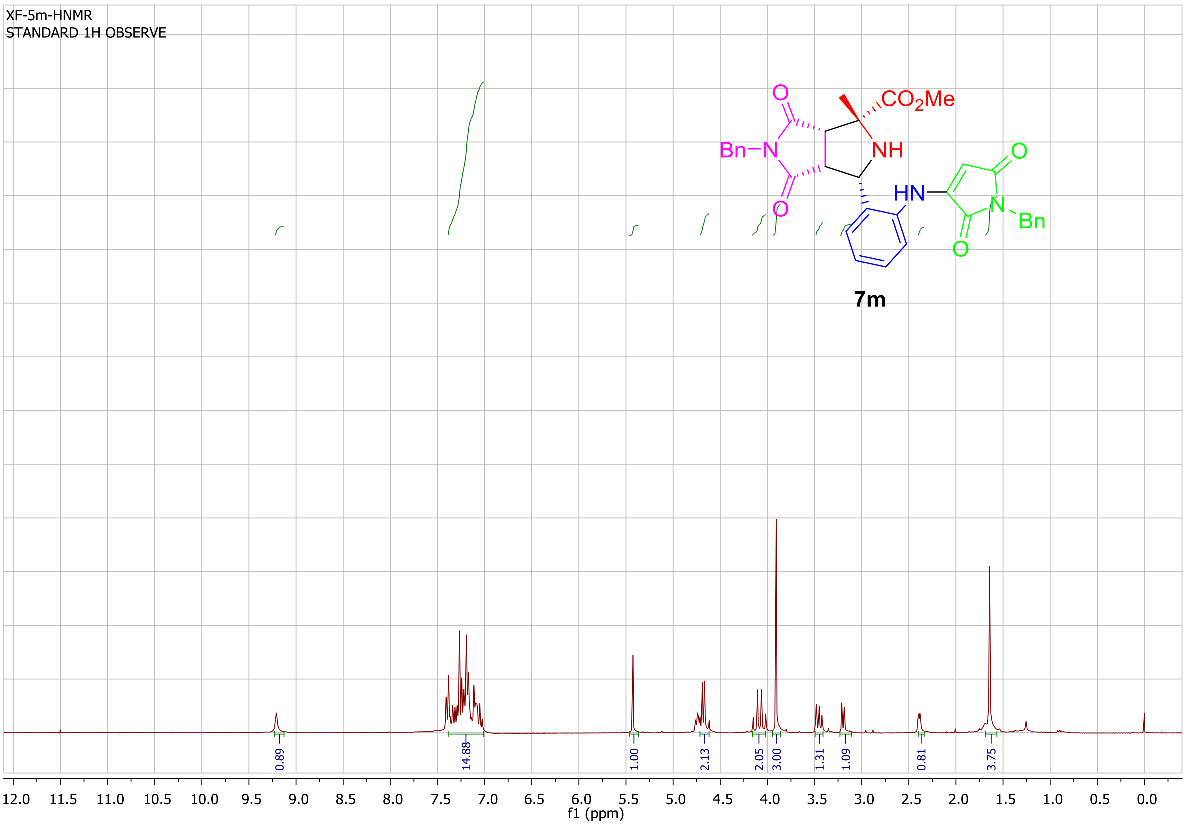
XF-5m-HNMR
STANDARD 1H OBSERVE
CO2Me
Bn-N
NH
HN
N
Bn
O
7m
0.89
14.88
1.00
2.13
2.05
3.00
1.31
1.09
0.81
3.75
f1 (ppm)

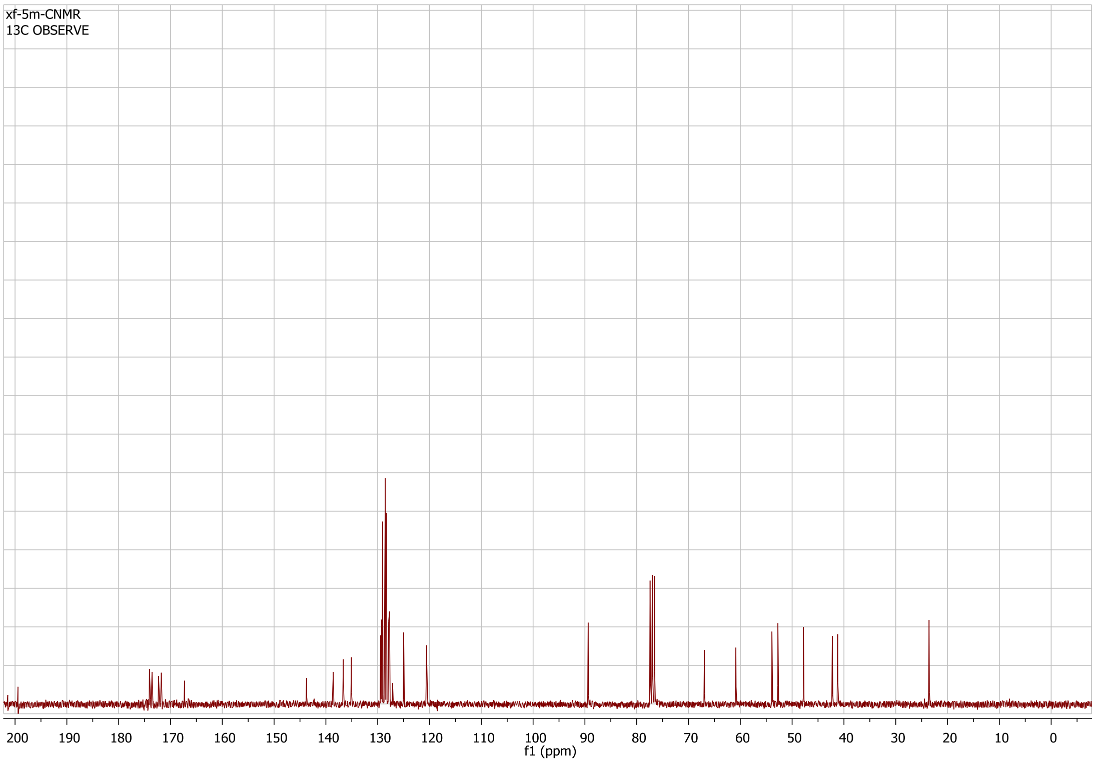
xf-5m-CNMR
13C OBSERVE
f1 (ppm)

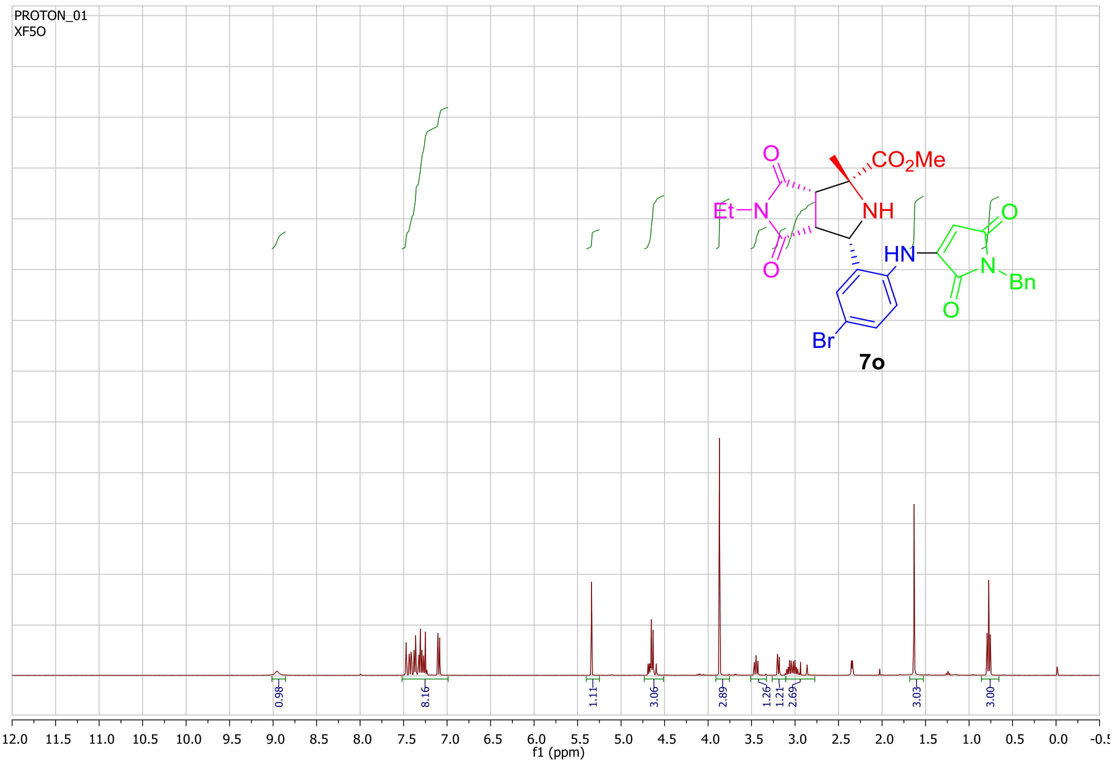
PROTON_01
XF5O
CO2Me
Et-N
NH
HN
N
Bn
Br
7o
f1 (ppm)

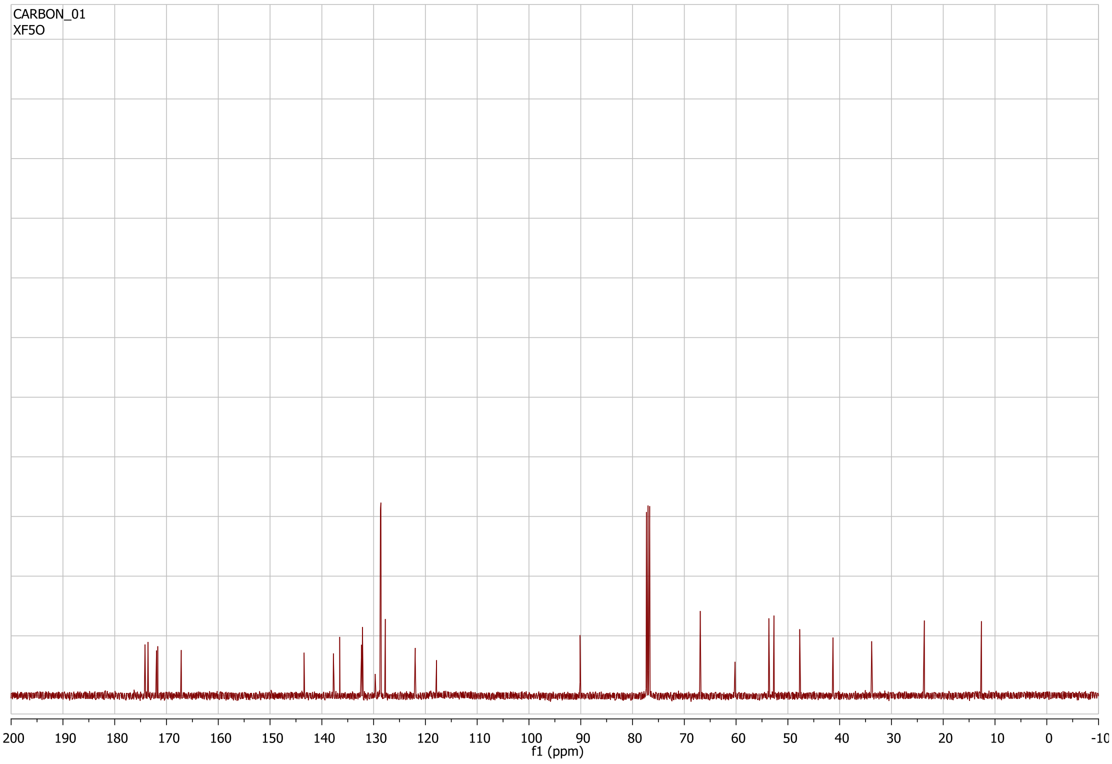
CARBON_01
XF5O
f1 (ppm)

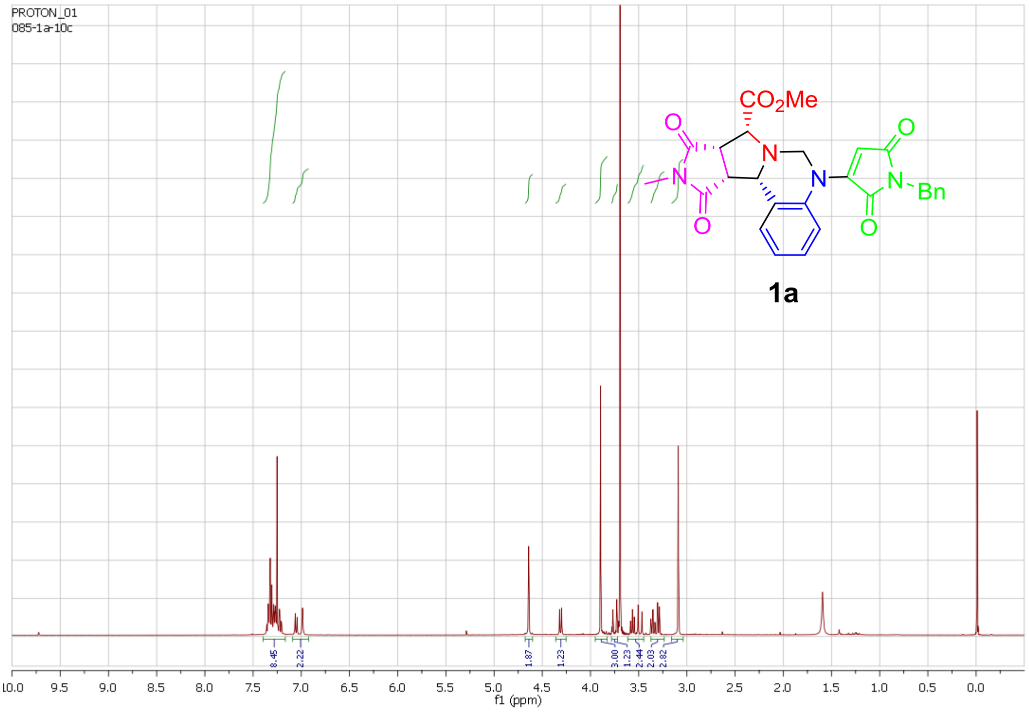
PROTON_01
085-1a-10c
CO2Me
N
O
Bn
1a
f1 (ppm)

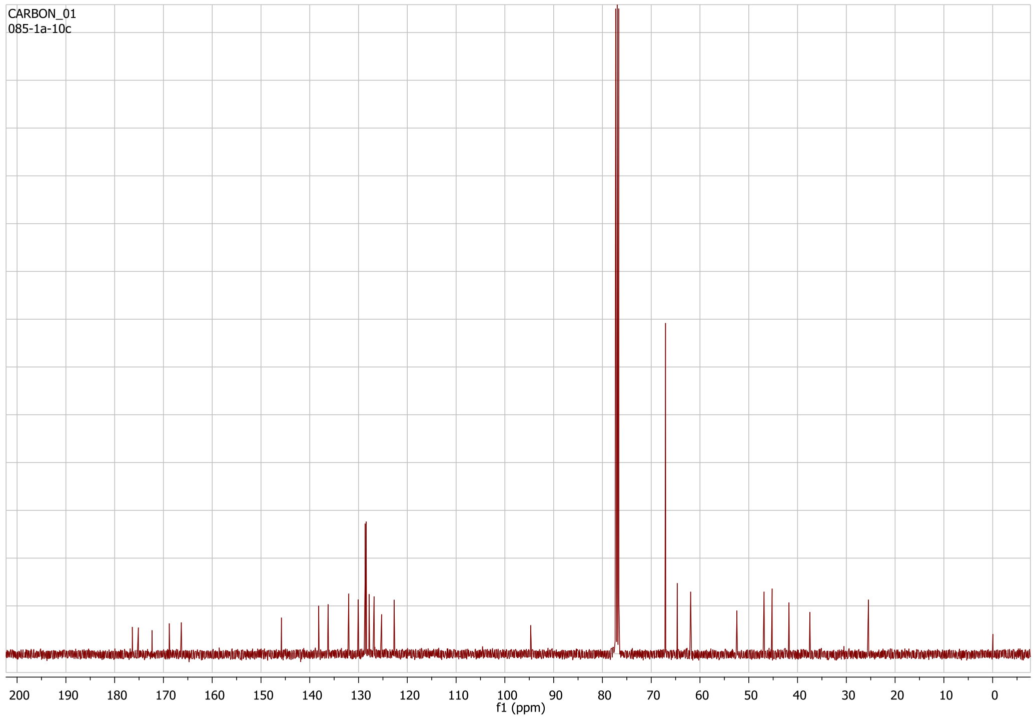
CARBON_01
085-1a-10c
f1 (ppm)

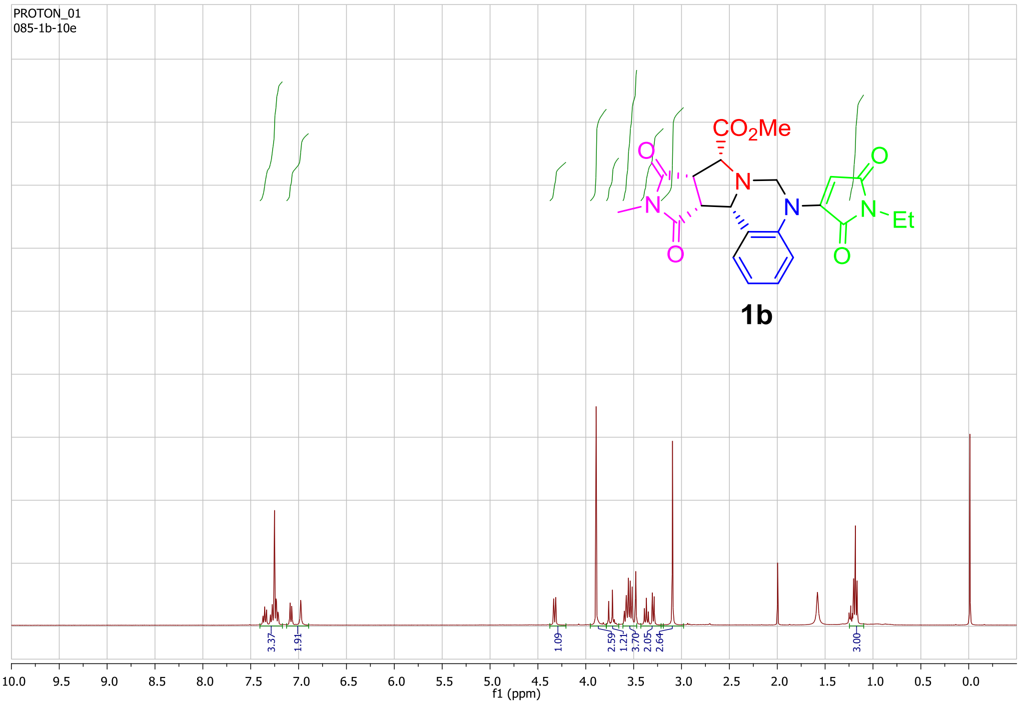
PROTON_01
085-1b-10e
CO2Me
O
N
N
N
O
N-Et
O
O
1b
3.37
1.91
1.09
2.59
1.21
3.70
2.05
2.64
3.00
f1 (ppm)

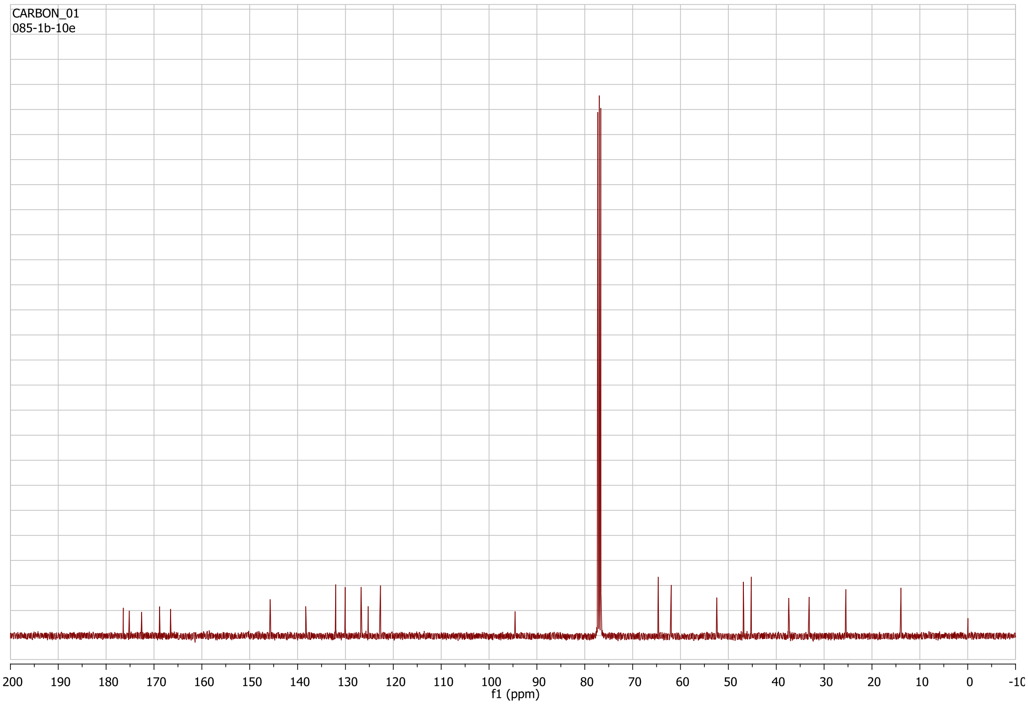
CARBON_01
085-1b-10e
f1 (ppm)

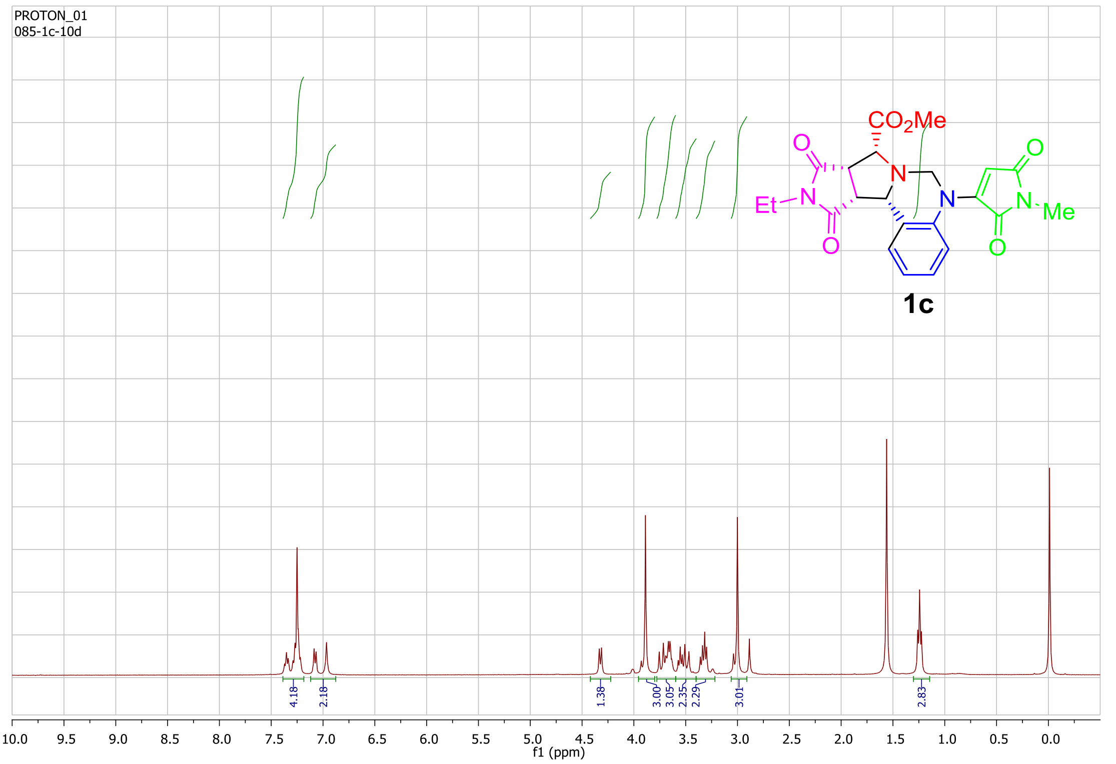
PROTON_01
085-1c-10d
CO2Me
O
Et
N
O
N
N
O
N
Me
O
1c
4.18
2.18
1.38
3.00
3.05
2.35
2.29
3.01
2.83
10.0 9.5 9.0 8.5 8.0 7.5 7.0 6.5 6.0 5.5 5.0 4.5 4.0 3.5 3.0 2.5 2.0 1.5 1.0 0.5 0.0
f1 (ppm)

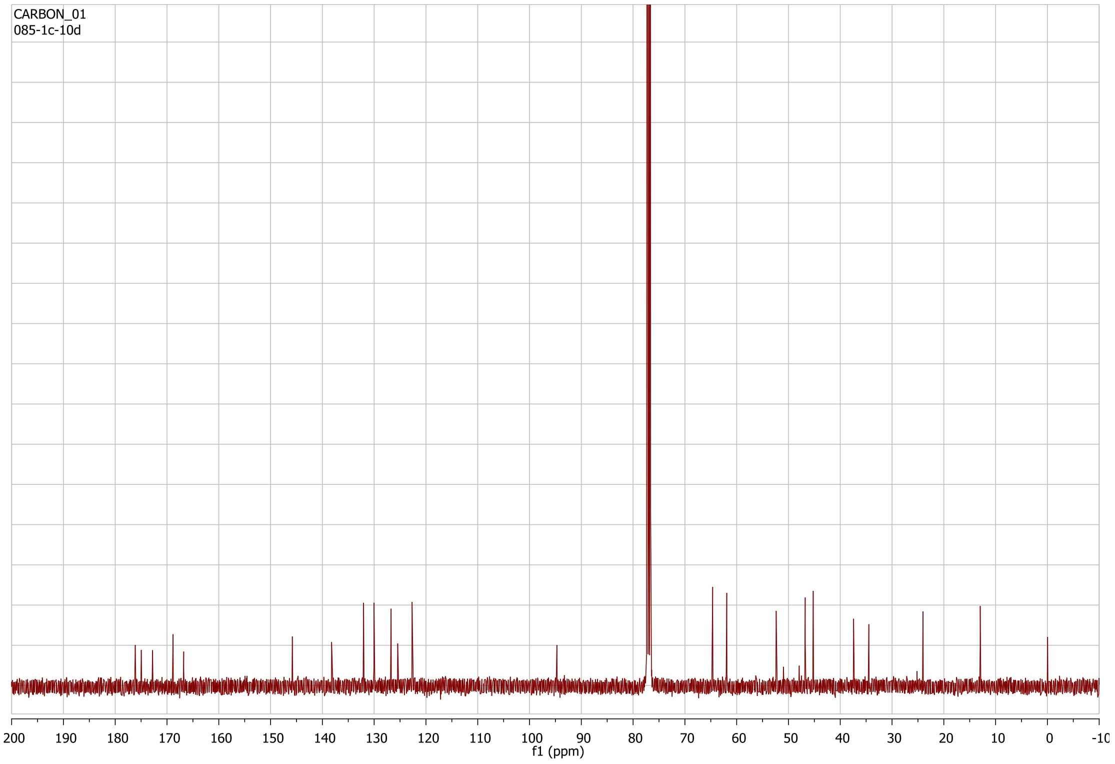
CARBON_01
085-1c-10d
200 190 180 170 160 150 140 130 120 110 100 90 80 70 60 50 40 30 20 10 0 -10
f1 (ppm)

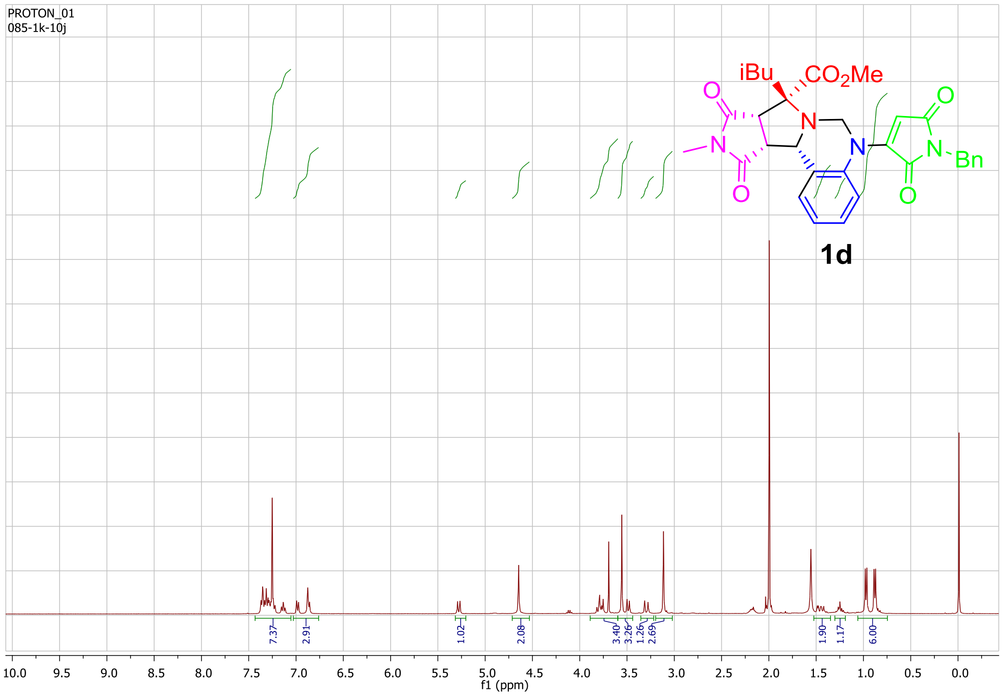
PROTON_01
085-1k-10j
iBu
CO2Me
O
N
N
N
O
N-Bn
O
O
1d
7.37
2.91
1.02
2.08
3.40
3.26
1.26
2.69
1.90
1.17
6.00
10.0 9.5 9.0 8.5 8.0 7.5 7.0 6.5 6.0 5.5 5.0 4.5 4.0 3.5 3.0 2.5 2.0 1.5 1.0 0.5 0.0
f1 (ppm)

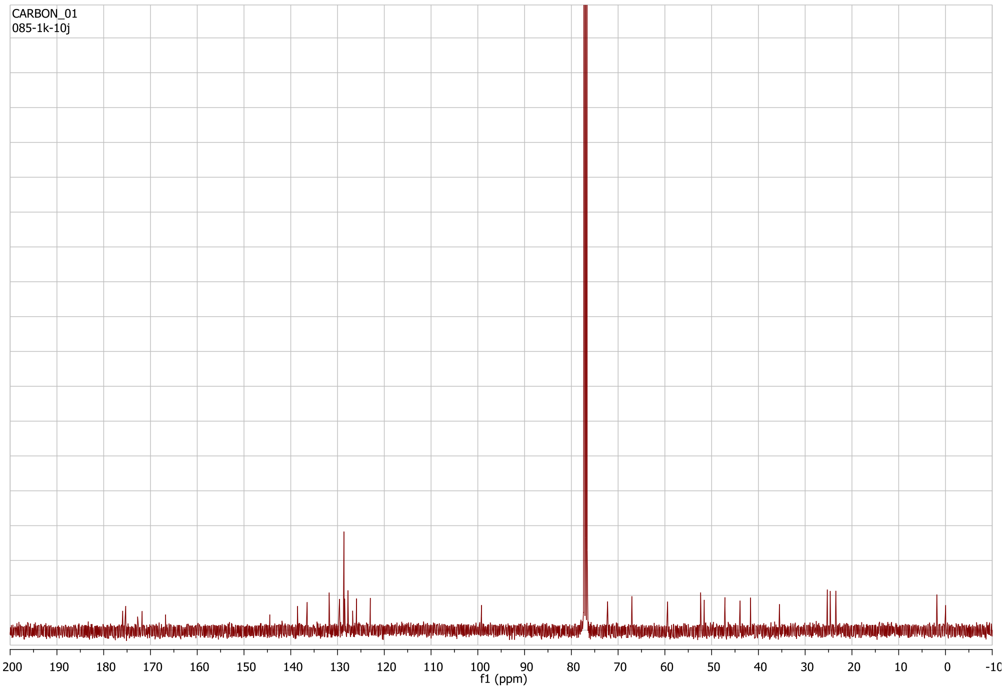
CARBON_01
085-1k-10j
200 190 180 170 160 150 140 130 120 110 100 90 80 70 60 50 40 30 20 10 0 -10
f1 (ppm)

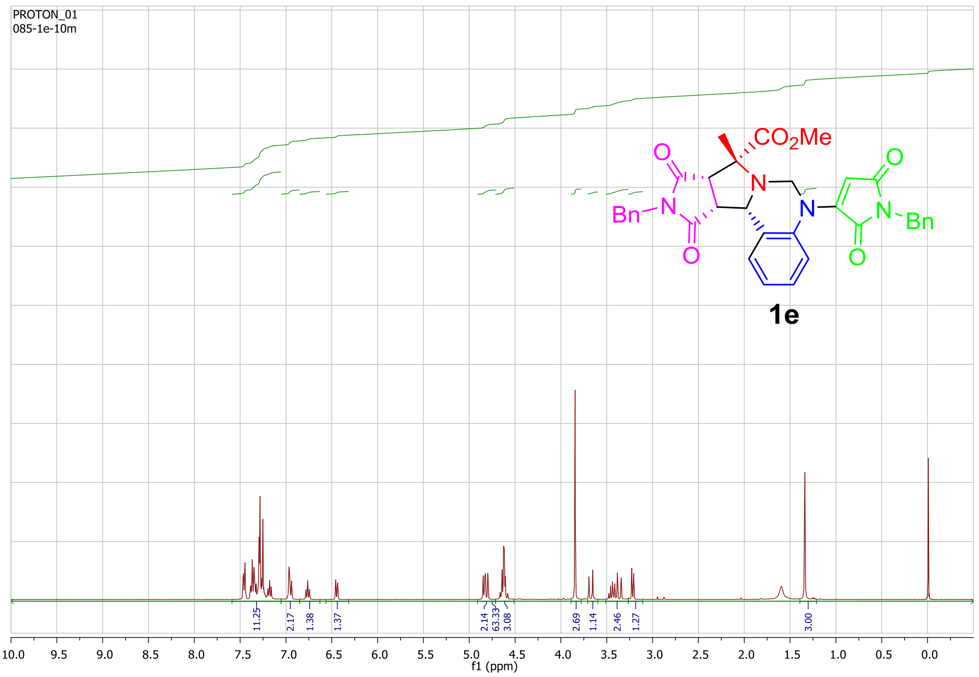
PROTON_01
085-1e-10m
CO2Me
Bn
N
O
1e
11.25
2.17
1.38
1.37
2.14
63.39
3.08
2.69
1.14
2.46
1.27
3.00
10.0 9.5 9.0 8.5 8.0 7.5 7.0 6.5 6.0 5.5 5.0 4.5 4.0 3.5 3.0 2.5 2.0 1.5 1.0 0.5 0.0
f1 (ppm)

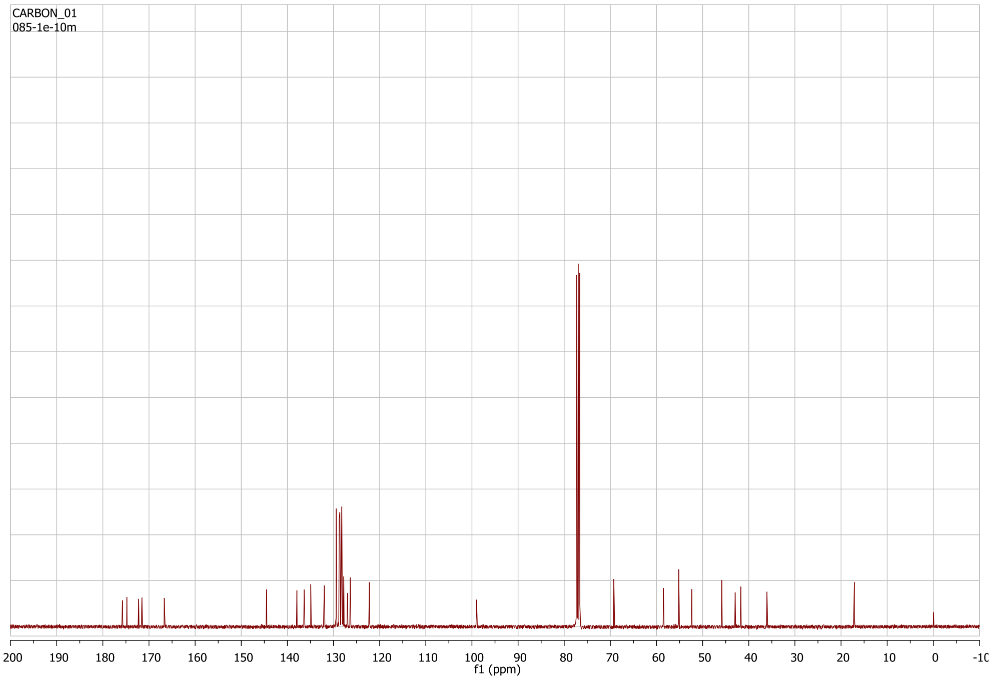
CARBON_01
085-1e-10m
200 190 180 170 160 150 140 130 120 110 100 90 80 70 60 50 40 30 20 10 0
f1 (ppm)

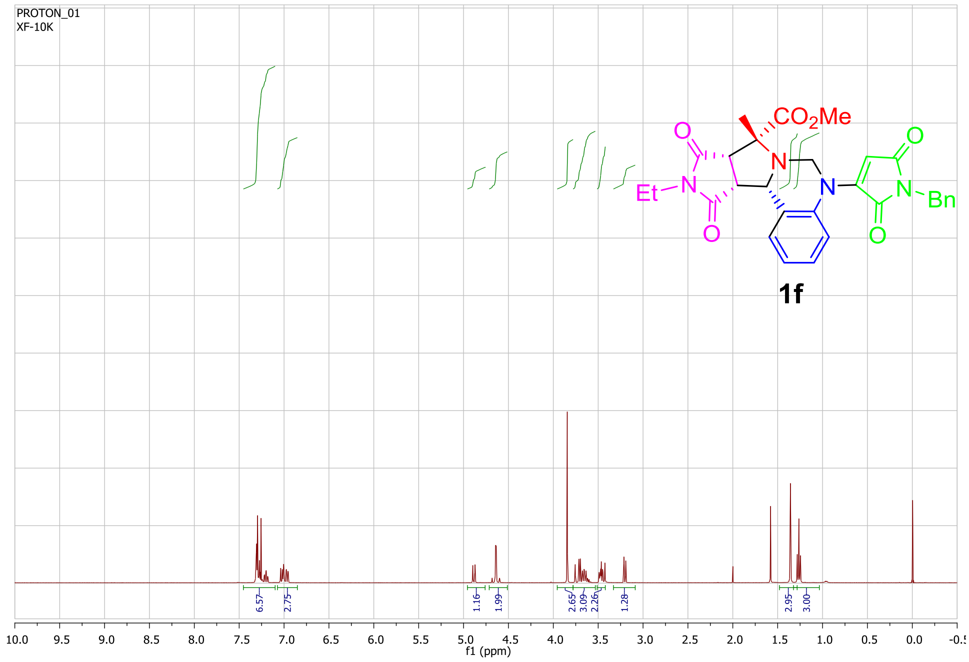
PROTON_01
XF-10K
CO2Me
Et
N
O
Bn
1f
6.57
2.75
1.16
1.99
2.65
3.09
2.26
1.28
2.95
3.00
10.0 9.5 9.0 8.5 8.0 7.5 7.0 6.5 6.0 5.5 5.0 4.5 4.0 3.5 3.0 2.5 2.0 1.5 1.0 0.5 0.0
f1 (ppm)

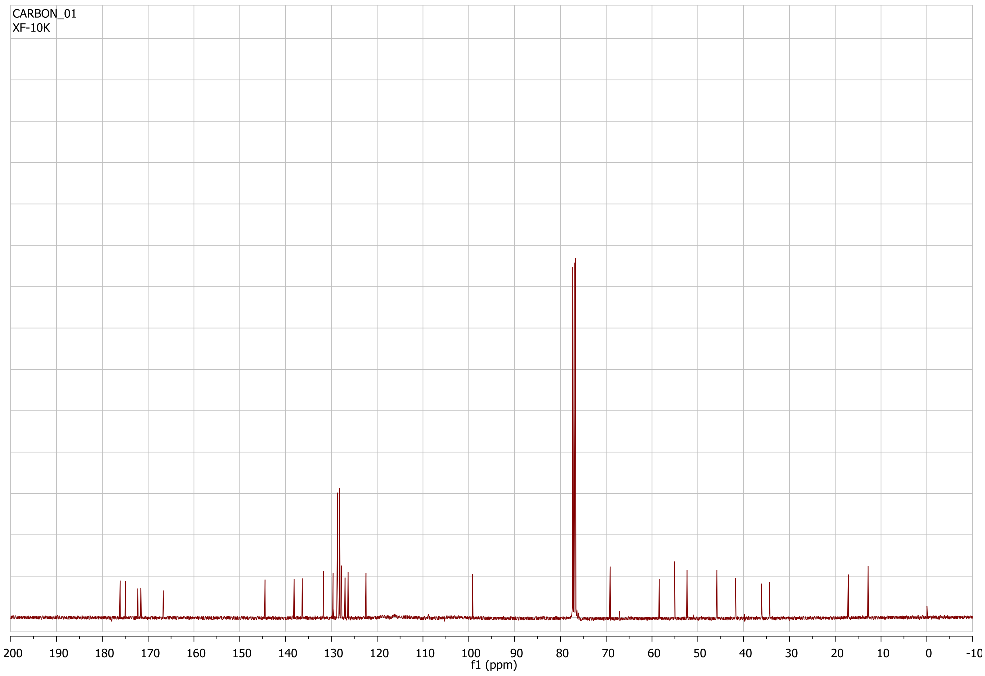
CARBON_01
XF-10K
200 190 180 170 160 150 140 130 120 110 100 90 80 70 60 50 40 30 20 10 0
f1 (ppm)

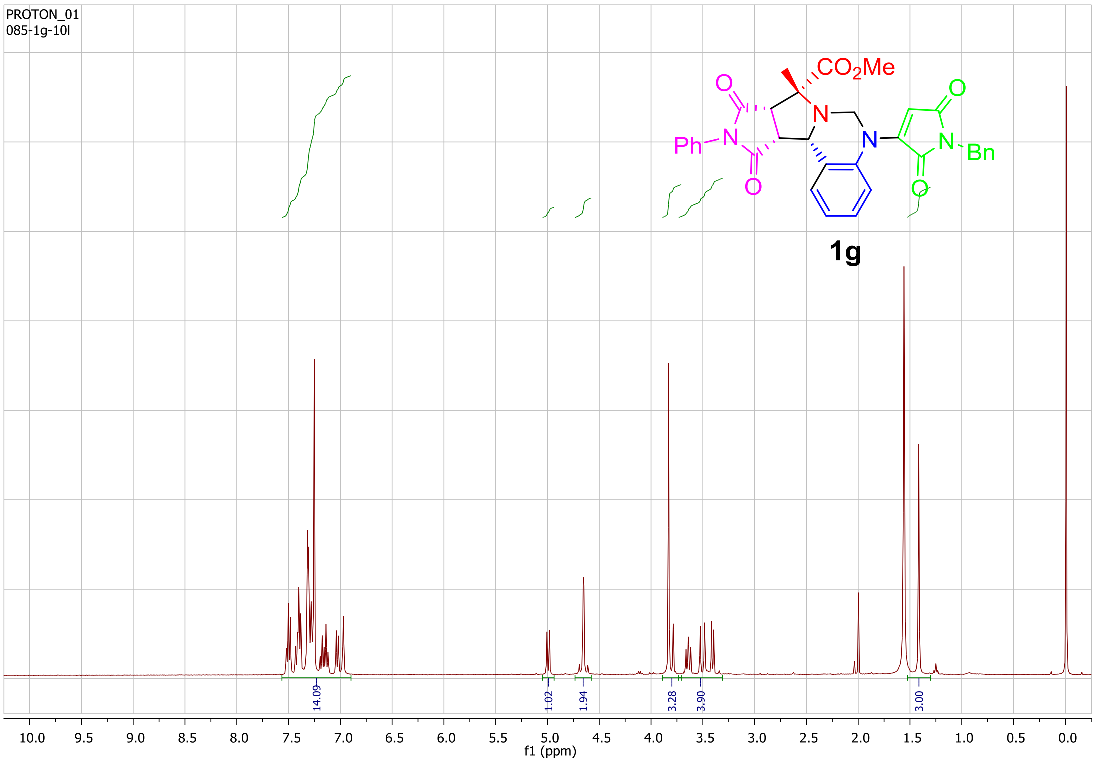
PROTON_01
085-1g-10l
CO2Me
O
N
Ph
N
O
N
O
N
Bn
O
1g
14.09
1.02
1.94
3.28
3.90
3.00
10.0
9.5
9.0
8.5
8.0
7.5
7.0
6.5
6.0
5.5
5.0
4.5
4.0
3.5
3.0
2.5
2.0
1.5
1.0
0.5
0.0
f1 (ppm)

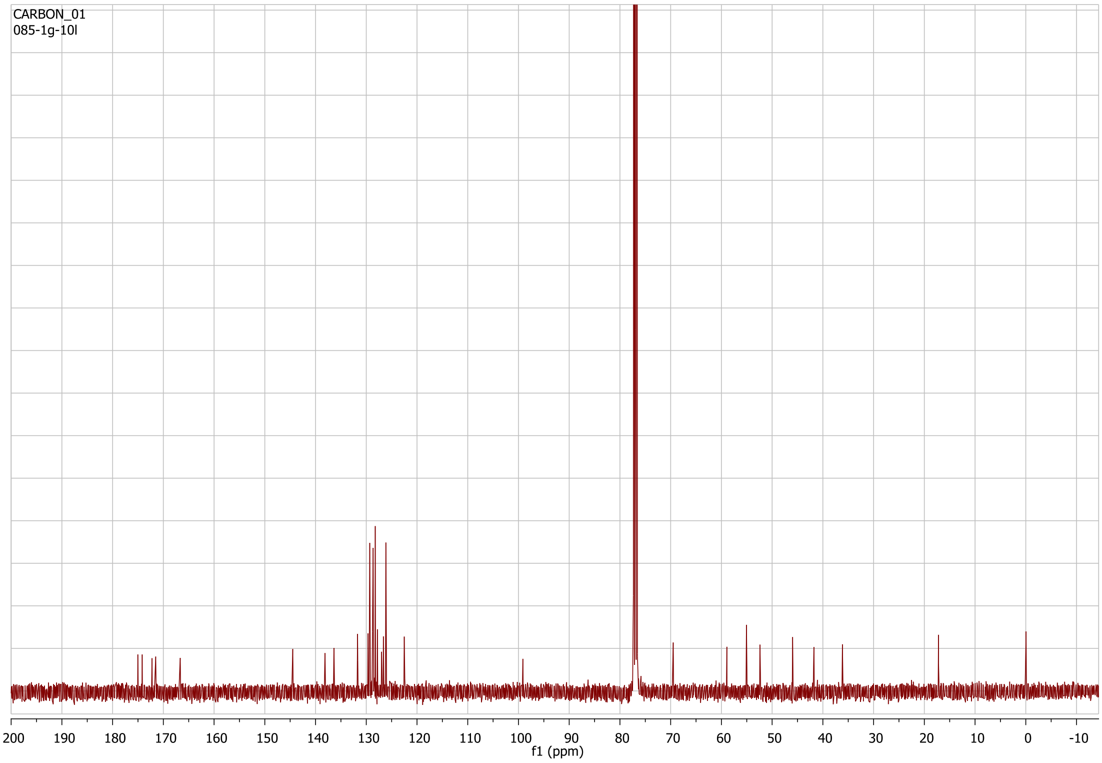
CARBON_01
085-1g-10l
200
190
180
170
160
150
140
130
120
110
100
90
80
70
60
50
40
30
20
10
0
-10
f1 (ppm)

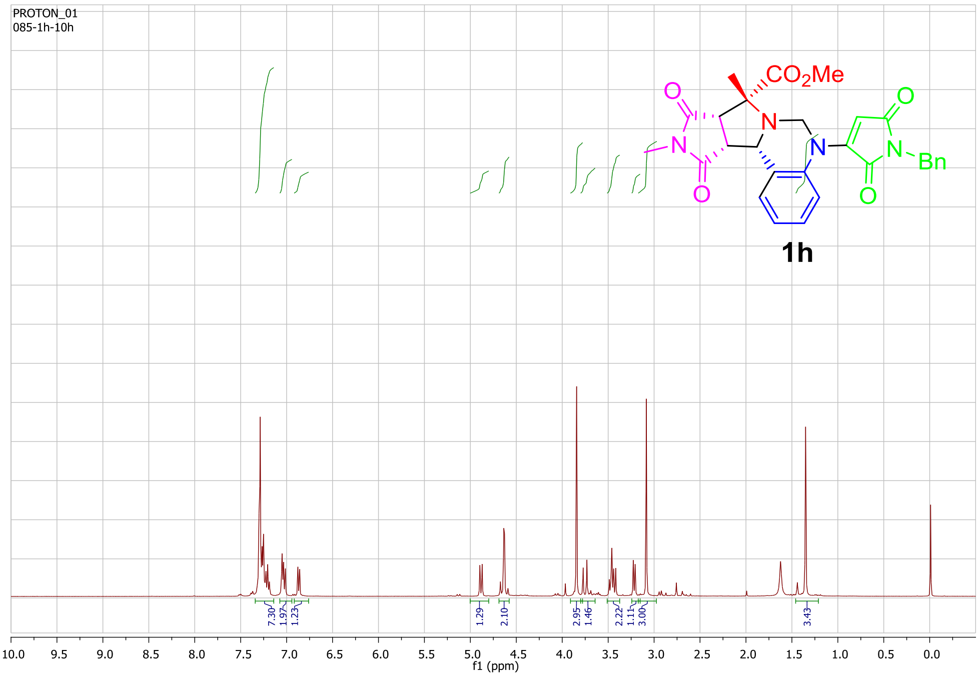
PROTON_01
085-1h-10h
CO2Me
O
N
N
N
O
O
O
Bn
1h
7.30
1.97
1.23
1.29
2.10
2.95
1.46
2.22
1.11
3.00
3.43
f1 (ppm)

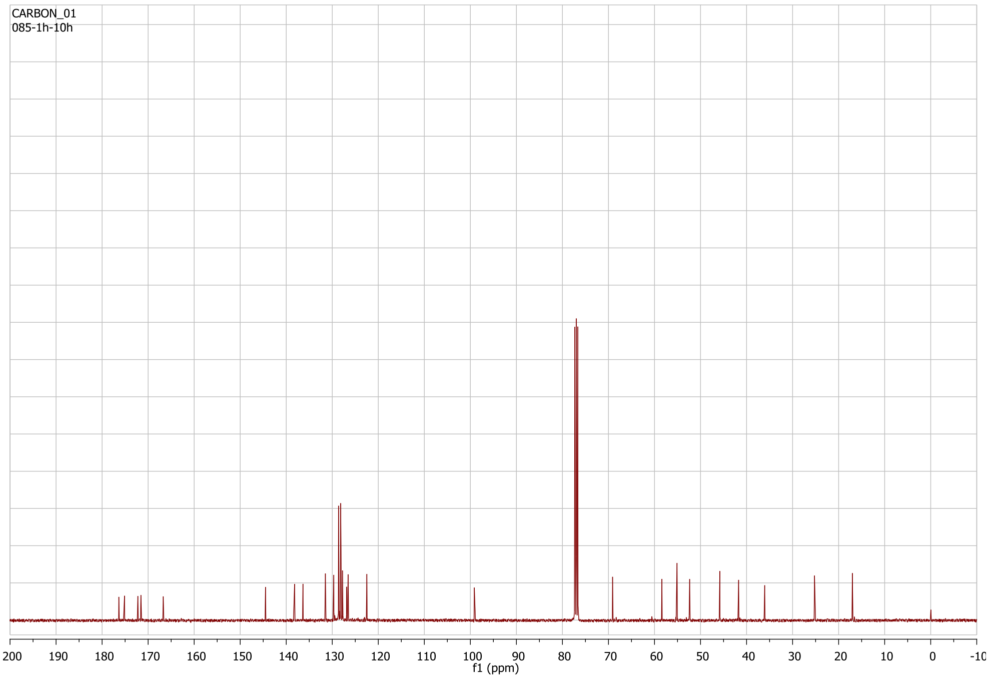
CARBON_01
085-1h-10h
f1 (ppm)

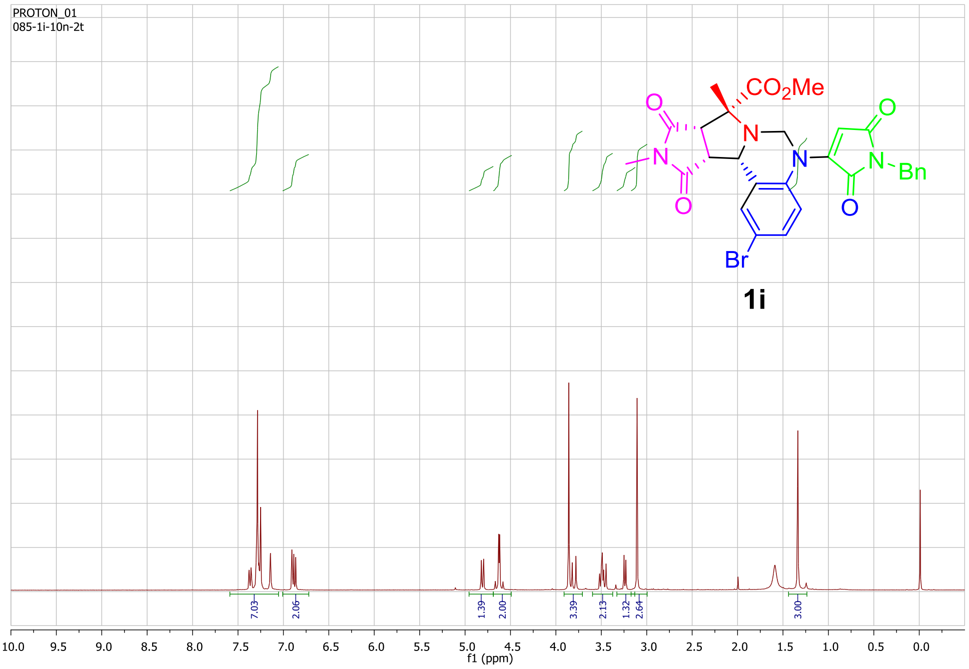
PROTON_01
085-1i-10n-2t
CO2Me
Bn
Br
1i
7.03
2.06
1.39
2.00
3.39
2.13
1.32
2.64
3.00
f1 (ppm)

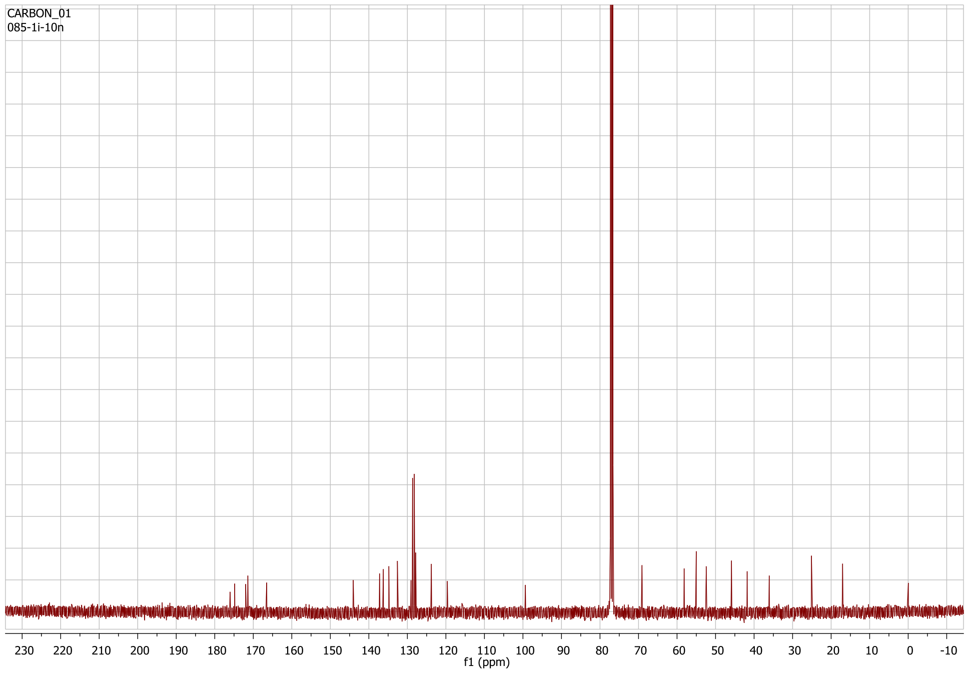
CARBON_01
085-1i-10n
f1 (ppm)

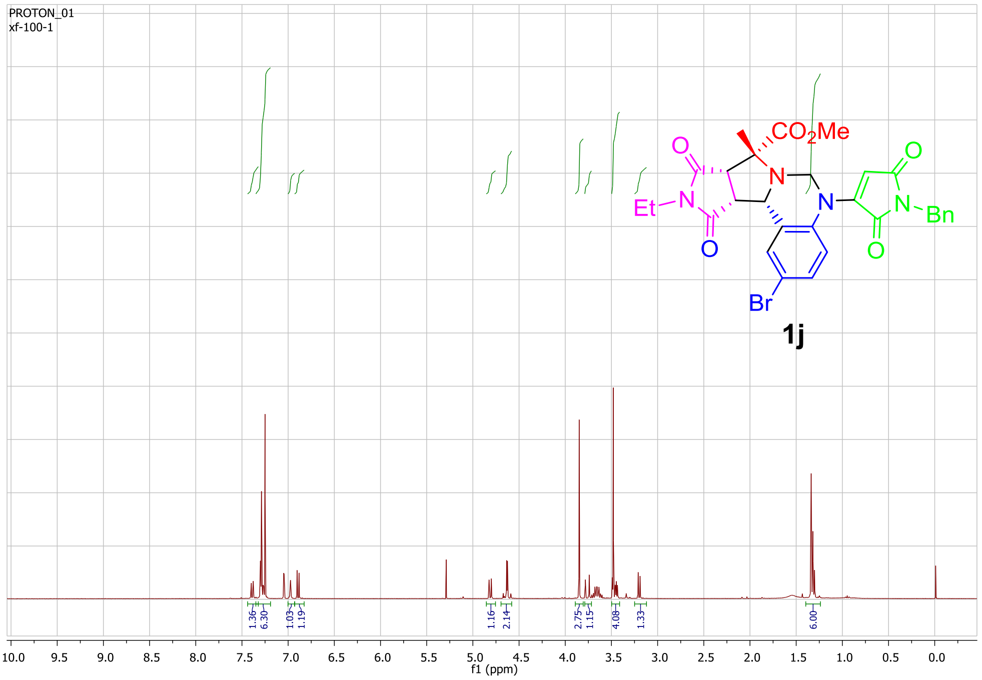
PROTON_01
xf-100-1
CO2Me
Et
N
O
Bn
Br
1j
f1 (ppm)

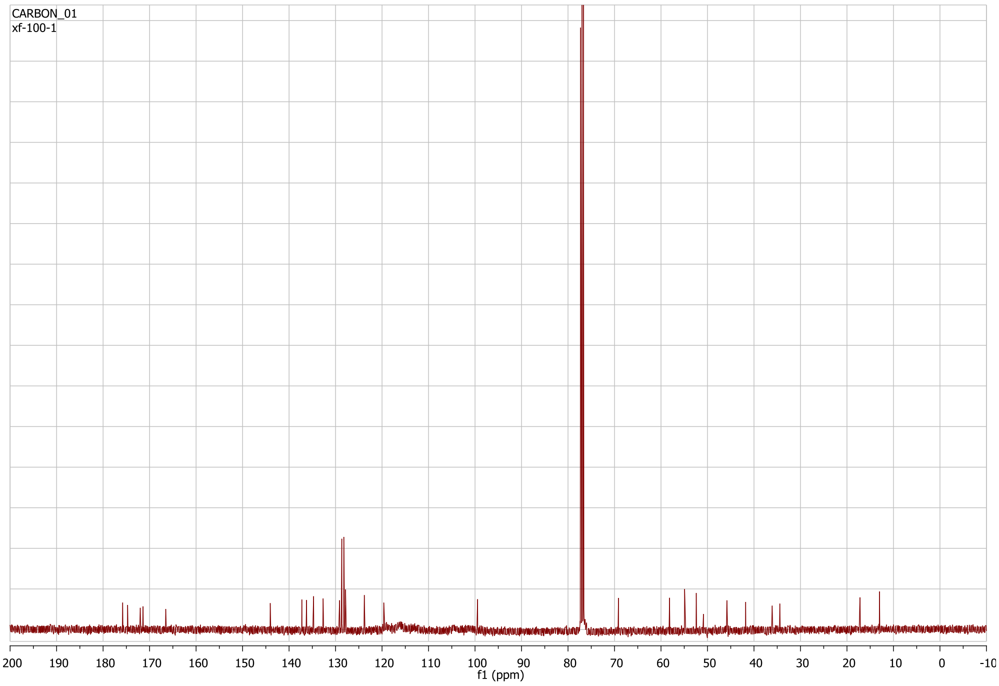
CARBON_01
xf-100-1
f1 (ppm)
